# Overexpression of *OsSAP16* Regulates Photosynthesis and the Expression of a Broad Range of Stress Response Genes in Rice (*Oryza sativa* L.)

**DOI:** 10.1371/journal.pone.0157244

**Published:** 2016-06-15

**Authors:** Fei Wang, Robert A. Coe, Shanta Karki, Samart Wanchana, Vivek Thakur, Amelia Henry, Hsiang-Chun Lin, Jianliang Huang, Shaobing Peng, William Paul Quick

**Affiliations:** 1 National Key Laboratory of Crop Improvement, MOA Key Laboratory of Crop Ecophysiology and Farming System in the Middle Reaches of the Yangtze River, College of Plant Science and Technology, Huazhong Agricultural University, Wuhan, Hubei province, 430070, China; 2 C4 Rice Center, International Rice Research Institute, Los Baños, Philippines; 3 Crop and Environmental Science Division, International Rice Research Institute, Los Baños, Philippines; Institute of Genetics and Developmental Biology, Chinese Academy of Sciences, CHINA

## Abstract

This study set out to identify and characterize transcription factors regulating photosynthesis in rice. Screening populations of rice T-DNA activation lines led to the identification of a T-DNA mutant with an increase in intrinsic water use efficiency (iWUE) under well-watered conditions. Flanking sequence analysis showed that the T-DNA construct was located upstream of LOC_Os07g38240 (*OsSAP16*) encoding for a stress-associated protein (SAP). A second mutant identified with activation in the same gene exhibited the same phenotype; expression of *OsSAP16* was shown to be enhanced in both lines. There were no differences in stomatal development or morphology in either of these mutants, although overexpression of *OsSAP16* reduced stomatal conductance. This phenotype limited CO_2_ uptake and the rate of photosynthesis, which resulted in the accumulation of less biomass in the two mutants. Whole transcriptome analysis showed that overexpression of *OsSAP16* led to global changes in gene expression consistent with the function of zinc-finger transcription factors. These results show that the gene is involved in modulating the response of rice to drought stress through regulation of the expression of a set of stress-associated genes.

## Introduction

Rice (*Oryza sativa*) is one of the most important food crops feeding nearly 50% of the world’s population. It serves as a model species for genomics research in grasses [1] and was the first crop plant to have the genome completely sequenced (International Rice Genome Sequencing Project 2005). This genomic information offers great potential to help understand how biological processes are regulated at the genetic level. It can also be used for crop improvement that aims to satisfy the increased demand for rice in a world undergoing rapid climate change. However, the majority of the approximately ~37,500 protein coding genes in the genome have no known function and 30% have no known homologs in *Arabidopsis* [2]. The challenge in the post-genomics era is to understand the function of these genes. One approach to help do this is to alter or eliminate the gene function with insertional mutagenesis using transfer DNA (T-DNA) where the inserted element acts a tag for gene identification [3–5]. Activation tagging is a modification of this method that uses T-DNA or transposable elements containing multimerized cauliflower mosaic virus (CaMV) 35S enhancers [6,7]. These cause transcriptional activation of nearby genes and gain of function mutations resulting in novel phenotypes associated with the gene function. This tagging system has been used extensively in *Arabidopsis* [8–13], petunia [14], periwinkle [15,16] and rice [17,18]. In our laboratory we have been screening two large T-DNA populations [18,19] for novel phenotypes associated with leaf morphology and photosynthetic performance [20] as part of the C_4_ Rice Project (http://c4rice.irri.org/). The work presented in this paper reports on a T-DNA mutant identified during these screens and the work undertaken to characterize the function of the gene activated in the mutants.

Transcription factors (TFs) are master regulators of gene expression. A single TF can control the expression of many target genes through the specific targeting non-coding DNA regions called *cis*-acting elements in the promoters of target genes. In this study, we identified a T-DNA insertion mutant in which the gene at LOC_Os07g38240 had been transcriptionally activated resulting in an increase in intrinsic water use efficiency (iWUE). This gene encodes for a C2H2-AN1 zinc finger transcription factor-*OsSAP16*, a member of an 18 gene family of Stress Associated Proteins (SAPs) in rice [21–22]. We hypothesize that *OsSAP16* controls photosynthesis through regulation of stomatal conductance via a set of stress response genes. A deeper understanding of the transcriptional regulatory networks underlying this response will lead to opportunities to improve stress tolerance at the molecular level.

## Results

### Identification of mutants with overexpression of *OsSAP16* and generation of homozygous plants

**Fig 1:**
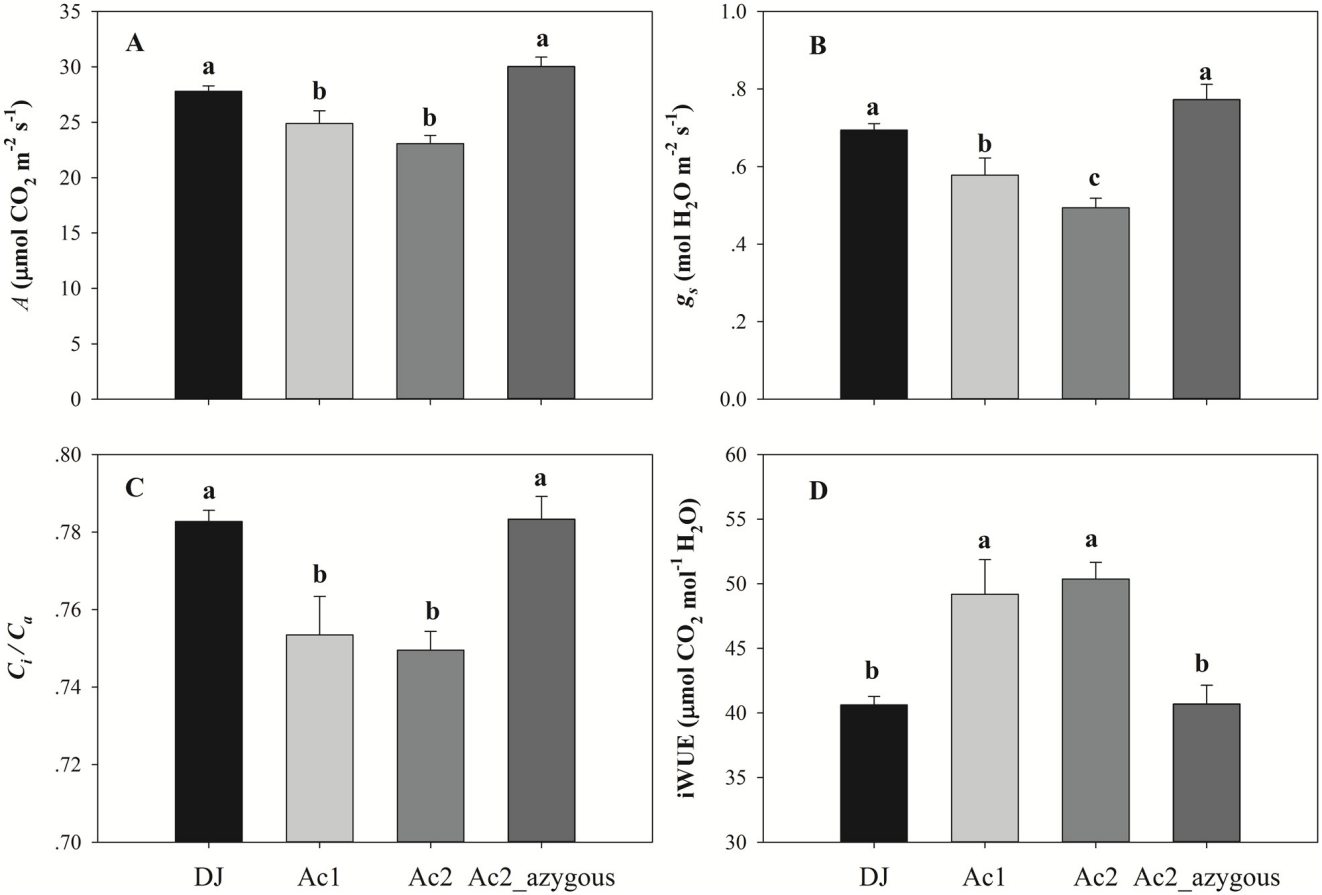
Instantaneous photosynthetic measurements. A, CO_2_ assimilation rate (*A*), B, stomatal conductance (*g*_*s*_,), C, intercellular CO_2_ / Ambient CO_2_ (*C*_*i*_*/C*_*a*_), and D, intrinsic water use efficiency (iWUE) measured at a CO_2_ concentration of 400 μmol CO_2_ mol^-1^ and a PPFD of 1500 μmol photons m^-2^ s^-1^ in Donjin (DJ), an azygous mutant line (Ac2_azygous) and two *OsSAP16* overexpression mutants (Ac1 and Ac2). Values are the average ± SE, where *n* = 51 (DJ), 33 (Ac1), 69 (Ac2) and 28 (Ac2_azygous). The mutant plants were in T_5_ generation for Ac1 and T_4_ generation for Ac2. Letters in each figure represent significant difference according to LSD at the 0.05 level.

**Fig 2:**
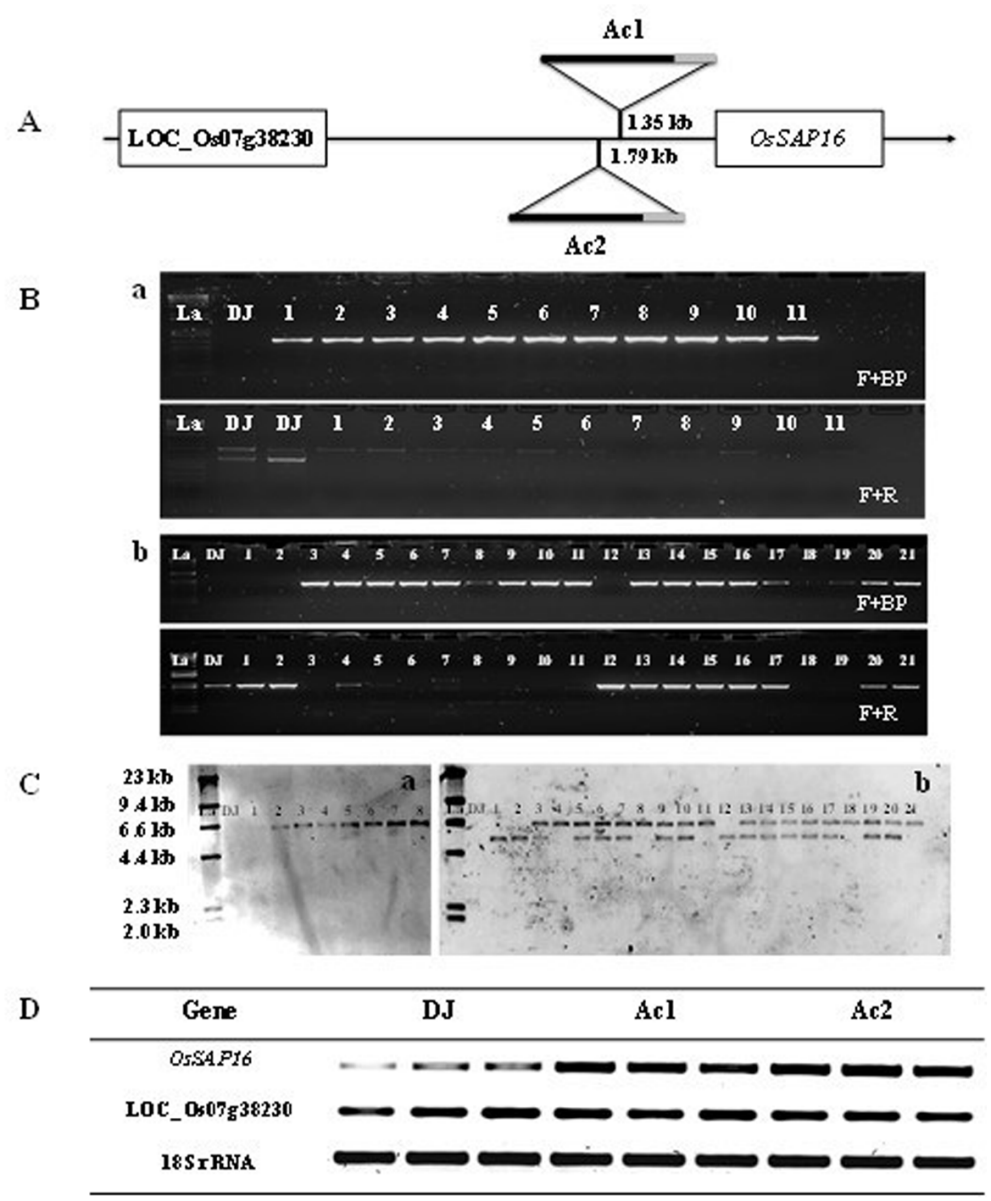
A, left border (grey color) of the T-DNA construct was inserted ~1.35kb upstream of LOC_Os07g38240 (OsSAP16) in mutant Ac1 and ~1.79kb in Ac2. The gene (LOC_Os07g38230) upstream of the insertion site encodes an expressed protein with unknown function. B, genotyping of the mutants to identify homozygous (HM) plants in T_4_ generation Ac1 mutants (a) and in T_3_ generation Ac2 mutants (b). Forward (F) and reverse (R) primers were designed to amplify the genomic sequence either side of the insertion site and the left boarder of the T-DNA construct (BP). The numbers indicate individual progenies for each mutant C, DNA blots show that Ac1 (a) carries a single copy of the construct and Ac2 (b) two. Untransformed rice control plants (DJ) are negative controls for the construct. La is the abbreviation of ladder. D, RT-PCR shows that the expression of LOC_Os07g38240 is higher in T_5_ generation Ac1 mutants and in T_4_ generation Ac2 mutants compared to T_5_ generation DJ, while the expression of LOC_Os07g38230 and the 18s RNA housekeeping gene remains unchanged.

Mutant Ac1 was identified from an instantaneous photosynthetic screen at a CO_2_ concentration of 400 ppm and a PPFD of 1500 μmol photos m^-2^ s^-1^. The photosynthetic phenotype of this mutant was characterized by reduced CO_2_ assimilation rates (*A*: Fig 1A), stomatal conductance (*g*_*s*_; Fig 1B), *C*_*i*_*/C*_*a*_ (Fig 1C) and a significantly increased iWUE (Fig 1D) compared to the azygous and wild type line. Analysis of the T-DNA flanking sequences from RiceGE (http://signal.salk.edu/cgi-bin/RiceGE/) showed that the construct was located 1.35 kb upstream of LOC_Os07g38240 (Fig 2A). A second mutant, Ac2 with an insertion site 1.79 kb upstream of this gene, exhibited a similar phenotype (Fig 1A).

The gene at LOC_Os07g38240 (*OsSAP16*) encodes a stress-associated protein (SAP) containing two conserved AN1-C2H2 zinc finger (ZnF) domains and one A20 domain (S1A and S1B Fig). The gene is highly expressed in leaf mesophyll and stomata cells, apical and axillary meristems of shoots, metaxylem in the root tip and endodermis cells in the root maturation zone (S2 Fig). In rice *OsSAP16* is a member of an 18-gene family with close homology to *ZmSAP110* from maize and *AtSAP11*, *14* and *13* from *Arabidopsis* (S1C Fig).

Homozygous (HM) progenies were identified for both mutants together with azygous plants for mutant Ac2 (Ac2_azygous; Fig 2B). DNA blot showed that progenies of mutant Ac1 contained a single copy of the T-DNA and Ac2 two copies (Fig 2C). RT-PCR showed that *OsSAP16* was significantly overexpressed in both the mutants, while the expression of the 18S rRNA housekeeping gene and LOC_Os07g38230, a gene with unknown function located downstream of the site of insertion remained unchanged (Fig 2D).

### *OsSAP16* overexpression mutants had higher iWUE

**Fig 3:**
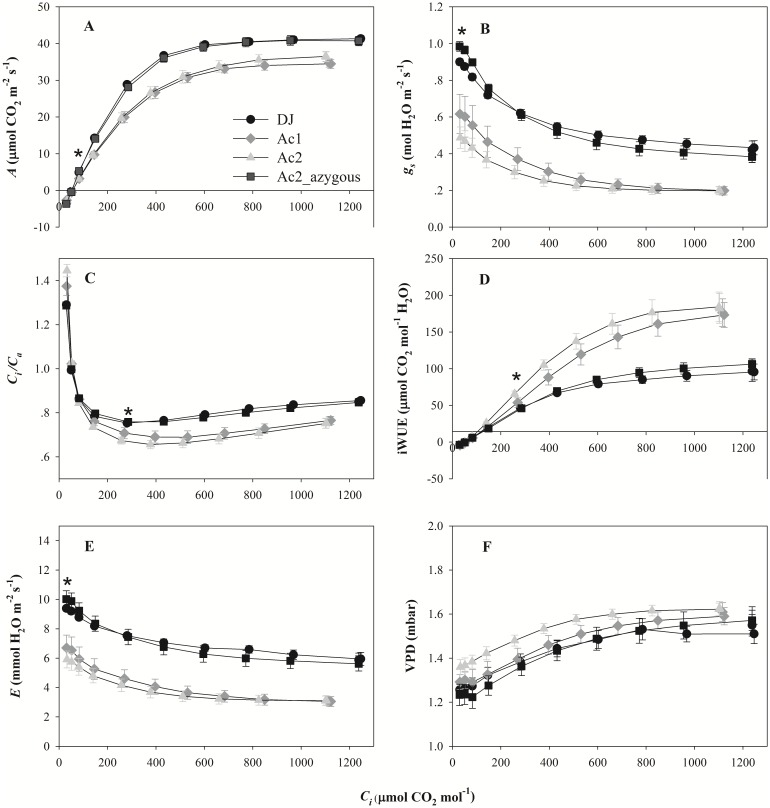
The response of CO_2_ assimilation to CO_2_ concentration in Donjin (DJ), an azygous mutant line (Ac2_azygous) and two *OsSAP16* overexpression mutants (Ac1 and Ac2). A, CO_2_ response curve of CO_2_ assimilation rate (*A*), B, stomatal conductance (*g*_*s*_), C, intercellular CO_2_ / Ambient CO_2_ (*C*_*i*_*/C*_*a*_), D, intrinsic water use efficiency (iWUE), E, transpiration rate (*E*), and F, vapor pressure deficit (VPD) measured at a PPFD of 1500 μmol photos m^-2^ s^-1^ Values are the average ± SE of one leaf from eight plants per line. The mutant plants were in the T_5_ generation for Ac1 and T_4_ generation for Ac2. Asterisk indicates the *C*_*i*_ at which the values for the mutants are statistically significantly different to DJ.

To further characterize the photosynthetic phenotype of the mutants, CO_2_ (*A*-*C*_*i*_) and light response curves were measured. Photosynthetic phenotype observed in the *A-C*_*i*_ curves was in line with that in the initial screen. *A* in the mutants were significantly lower than DJ or Ac2_azygous above a *C*_*i*_ of ~100 μmol CO_2_ mol^-1^ (Fig 3A), while *g*_*s*_ responded to changes in *C*_*i*_ it was approximately 30% lower in the mutants (Fig 3B). This was associated with lower *C*_*i*_/*C*_*a*_ (Fig 3C), transpiration rates (*E*; Fig 3E) and higher iWUE (Fig 3D). There were no significant differences in VPD between the lines (Fig 3F). A significant positive correlation between *g*_*s*_ and *A* was found (R^2^ = 0.9973, *p* = 0.002). The carboxylation efficiency (CE) of both mutants was significantly lower and the CO_2_ compensation points (*Γ*) significantly higher than DJ and Ac2_azygous (S4A and S4B Fig).

**Fig 4:**
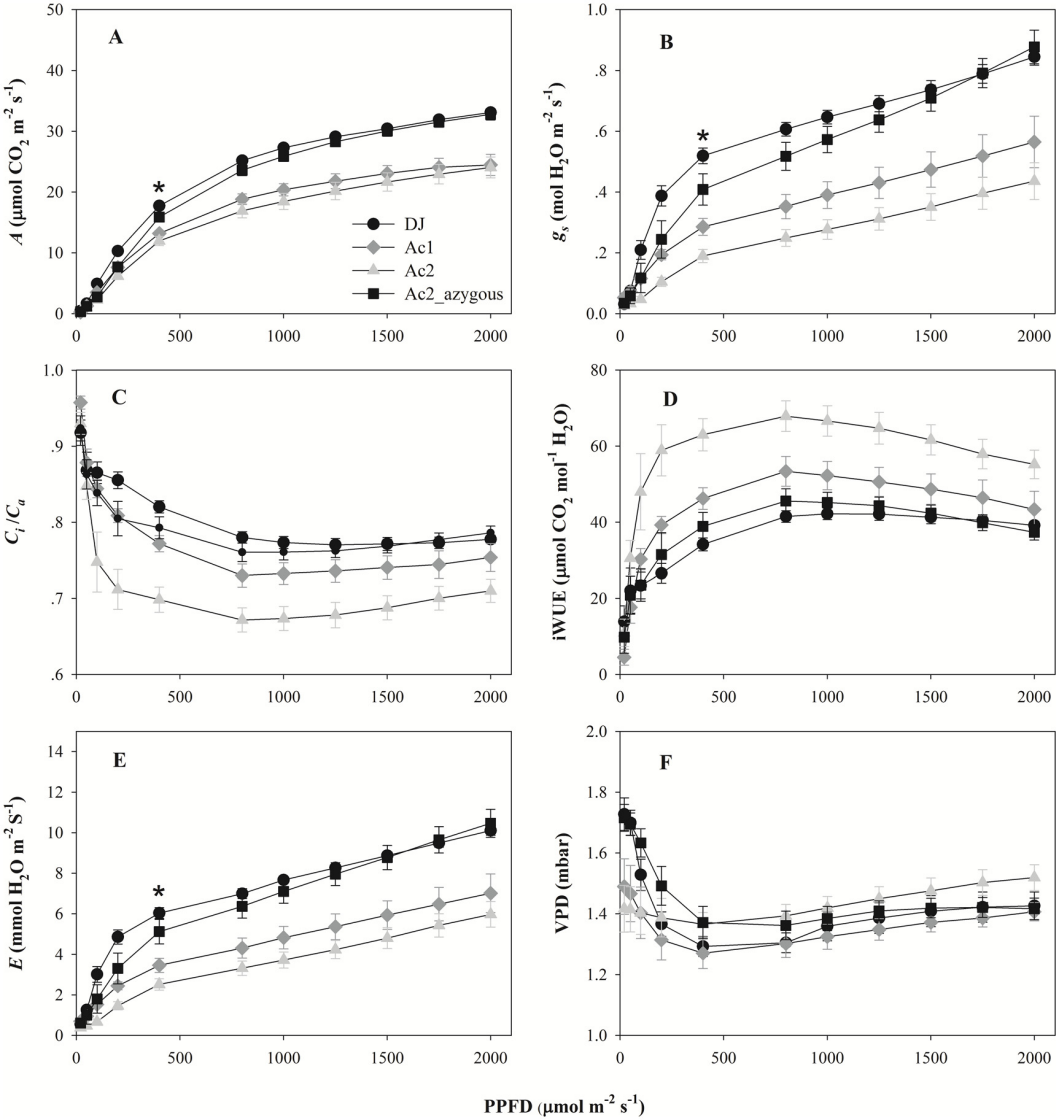
The response of CO_2_ assimilation to photosynthetic photon flux density (PPFD) in Donjin (DJ), an azygous mutant line (Ac2_azygous) and two *OsSAP16* overexpression mutants (Ac1 and Ac2). A, CO_2_ assimilation rate (*A*), B, stomatal conductance (*g*_*s*_), C, intercellular CO_2_ / Ambient CO_2_ (*C*_*i*_*/C*_*a*_), D, intrinsic water use efficiency (iWUE), E, transpiration rate (*E*), and F, vapor pressure deficit (VPD) measured at a CO_2_ concentration of 400 μmol CO_2_ mol^-1^. Values are the average ± SE of one leaf from eight plants per line. The mutant plants were in the T_5_ generation for mutant Ac1 and T_4_ generation for mutant Ac2. Asterisks indicate that values of the two mutants were significantly lower than DJ and Ac2_azygous.

Mutants had lower rates of *A* than WT or azygous lines above a photon flux density (PPFD) of ~400 μmol m^-2^ s^-1^ (Fig 4A). iWUE was significantly higher in mutant Ac2 but not Ac1 (Fig 4D), the discrepancy in the response of the lines is due to significantly lower *C*_*i*_/*C*_*a*_ in Ac2.*φ* was significantly lower in both mutants compared to DJ, but only in mutant Ac2 when compared to Ac2_azygous (S4C Fig). No significant differences were observed in the leaf chlorophyll content between the lines (S5 Fig).

### *OsSAP16* overexpression mutants had altered leaf anatomy

*OsSAP16* mutants had a higher mesophyll cell number and smaller bundle sheath cell area (S2 Table). No significant differences were found in any of the other parameters measured. In addition, no qualitative differences were detected in stomatal density, shape, or size, except that epidermal cells were significantly shorter (S3 Table).

### *OsSAP16* overexpression mutants had reduced growth rates

Mutants were significantly shorter than DJ, and had lower total plant dry weight, shoot and root biomass (Table 1). No consistent differences in the root to shoot ratio, rate of water uptake or water use efficiency (WUE) were observed between the lines. Significant differences in root traits were observed with the mutants having a smaller and less extensive root architecture compared to DJ (S4 Table).

**Table 1:** Plant growth and water use of Dongjin and two *OsSAP16* overexpression mutants (Ac1 and Ac2) under two experimental treatments (30 DPG).

Treatment Line	Plant height (cm)	Total plant dry biomass (g)	Total dry shoot biomass (g)	Total dry root biomass (g)	Root:Shoot ratio	Water uptake (mL)	WUE (g mL^-1^)
DD							
DJ	35.5±1.3^a^	117.7±10.9^a^	97.3±9.6^a^	20.4±1.4^a^	0.213±0.01^a^	99.3±3.4^a^	1.26±0.08^a^
Ac1	25.3±1.0^c^	65.3±7.7^b^	53.3±6.4^b^	11.9±1.5^b^	0.227±0.02^a^	80.7±4.3^b^	0.77±0.07^b^
Ac2	28.6±1.3^b^	86.5±7.1^b^	69.5±6.9^b^	16.9±1.6^a^	0.253±0.03^a^	86.7±3.0^b^	0.95±0.05^b^
WW							
DJ	46.9±0.8^a^	233.5±9.9^a^	207.5±9.1^a^	26.0±0.8^a^	0.122±0.01^a^	259.5±10.8^a^	0.88±0.03^a^
Ac1	39.2±0.7^b^	132.1±10.0^c^	117.8±10.0^c^	14.3±1.4^c^	0.126±0.01^a^	227.0±7.6^b^	0.57±0.03^b^
Ac2	41.9±1.1^b^	188.8±12.9^b^	167.2±12.9^b^	21.6±1.5^b^	0.134±0.02^a^	260.8±11.3^a^	0.73±0.04^ab^
ANOVA							
Tre.	***	***	***	**	***	***	***
Line	***	***	***	***	ns	***	*
Tre.*Line	ns	ns	ns	ns	ns	ns	ns

WW: well watered treatment, DD: dry down treatment, ANOVA: Analysis of Variance, WUE: water use efficiency. Data shown here are the average ± SE of five plants. Different letters indicate statistically significant differences within the treatments at the 0.05 level. Asterisks represent the significant differences amongst lines, water treatments and lines×water treatment.

*: p<0.05,

**: p<0.01,

***: p<0.001,

ns: not significant.

### *OsSAP16* controls the expression of stress response genes

**Fig 5:**
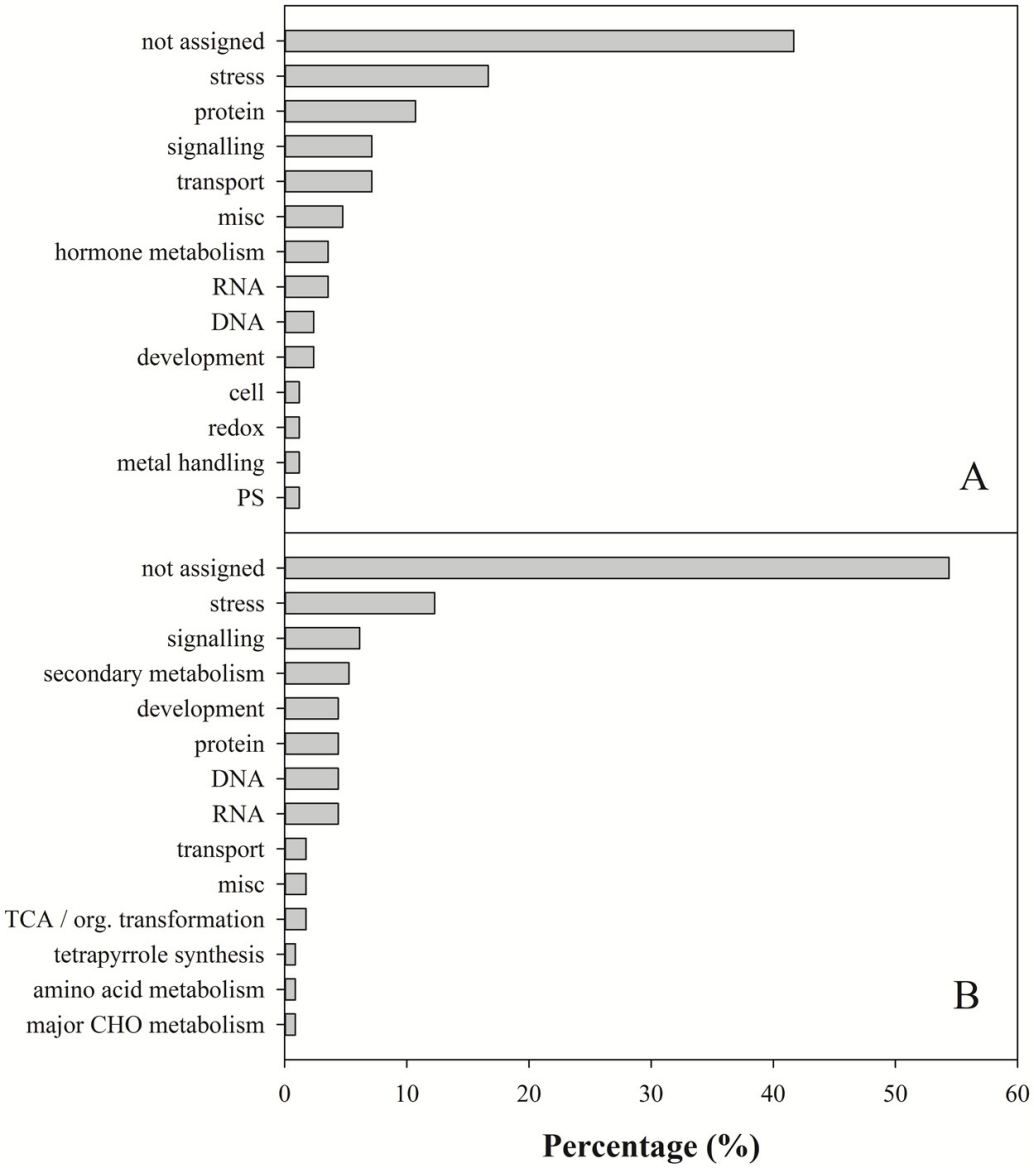
Functional categorization of significantly down- (A) and up- (B) regulated genes in two *OsSAP16* overexpression mutants (Ac1 and Ac2) compared to the wildtype Dongjin. MapMan was used to classify the genes into different biological processes.

As zinc finger proteins are generally known to be transcription factors involved in gene network regulation, RNA sequencing (RNA-Seq) was performed to identify the genes affected by overexpression of *OsSAP16*. Significant differentially expressed genes were identified in the mutants using three different DE analysis tools. A total of 84 overlapping genes were found to be significantly downregulated and 114 significantly upregulated in both mutants (S6 Fig and S5 Table). These were classified into 14 functional groups using Mapman visualization software (Fig 5). Of the genes identified 40% had no known biological function (Fig 5), whereas those associated with stress comprised the largest functional category of both the significantly up- (12%) and down- (17%) regulated genes. The functions of these genes were visualized by mapping them on to the metabolic pathways to which they belong (S7A Fig). For the up-regulated genes, there were 12 genes encoding pathogenesis-related (PR) proteins, 8 genes encoding signaling proteins, 6 genes encoding protein in secondary metabolism and a single gene encoding a myb transcription factor, peroxidase and a beta-gluconase (S7A Fig). For the downregulated genes, there were 12 genes encoding PR-proteins, 7 involved in signaling, 5 involved in proteolysis, 2 involved in ethylene signaling, 1 gene involved in ABA signaling, 1 encoding a bZIP transcription factor, and 1 encoding a gene regulating the redox state (S7A Fig).

Genes involved in primary metabolism were mostly up-regulated with the exception of those involved in photorespiration (S7B Fig). Approximately 5% of the upregulated genes identified were associated with secondary metabolism. Of these, 3 were involved in the production of phenolypropanoids, 3 with the production of lignins and lignans, and 1 each with the production of isoflavonoids, flavanols and alkaloid-like compounds (S7C Fig). The top ten genes with the highest levels of expression (6–9 fold increase) were either classed as protein kinases implicated in a diverse range of signaling processes or abiotic disease resistance proteins (S5 Table). GO enrichment analysis confirmed that the upregulated genes were principally involved in regulating the response to stress, were located in the plasma membrane and had kinase activity (S8C and S8D Fig).

### Expression of *OsSAP16* is upregulated in response to drought

**Fig 6:**
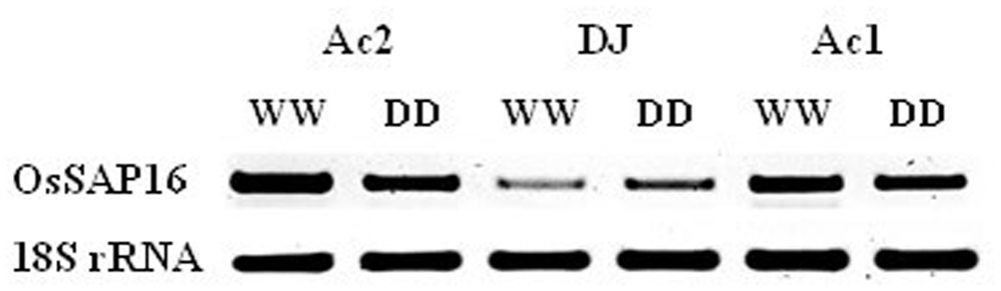
Expression of *OsSAP16* and 18S rRNA in the leaves of DJ, Ac1 and Ac2 under well watered (WW) and dry down (DD) conditions.

Plants were exposed to drought stress to test if *OsSAP16* plays a role in the response of rice to stress. *OsSAP16* expression was significantly upregulated in wild type plants but decreased in the mutants relative to the abundance under WW conditions (Fig 6). Despite this, *OsSAP16* expression remained several fold higher in the mutants compared to the wild type under both conditions.

The phenotype of plants under drought conditions was similar, with significant reductions in plant height, shoot biomass, root biomass, total plant dry biomass and water uptake relative to plants grown under WW conditions (Table 1). The root to shoot ratio and WUE increased in all lines. Similar phenotypic differences were observed between the mutants and DJ under drought (DD) conditions as observed under WW conditions. Mutants were significantly shorter, had lower shoot, root and total plant dry biomass, and they took up less water than DJ and had lower WUE (Table 1). Differences in root architecture were observed with the maximum root depth, number of root forks and percentage of lateral roots increasing and root diameter decreasing (S4 Table).

## Discussion

### Overexpression of *OsSAP16* leads to higher iWUE

In this study we have characterized the function of *OsSAP16*, a member of the 18-gene SAP gene family in rice [21]. Overexpression of *OsSAP16* in two separate T-DNA activation mutants resulted in a significant increase in iWUE. Our results show that this is due to a reduction in *g*_*s*_ rather than *A* (Figs 3 and 4). No significant difference in stomatal density or size in the mutants were found (Table 1), indicating that stomatal opening but not development has been altered in the mutants. In C_3_ species, it is well known that stomatal conductance is correlated with photosynthesis rate [23–25] and yield [26–28]. Consistent with this, a reduction in *g*_*s*_ restricts the rate of *A* and leads to a lower *E* in the mutants (Figs 3 and 4). This leads to a reduction in the growth rate of the mutants under well watered conditions (Table 1). However, this does not lead to a consistent reduction in water uptake or a WUE for biomass production (Table 1). This presumably reflects the difference between measurements made inside the cuvette of the gas exchange system, where VPD is constrained to be constant, and the natural environment. Outside of the cuvette, a decrease in *g*_*s*_ will lead to an increase in leaf temperature and an increase in the gradient driving transpiration. This would lead to higher rates of *E* in the mutants, despite lower rates of *g*_*s*_. In our previous study, higher transpiration rate is beneficial for high-temperature tolerance in rice [29].

*OsSAP16* overexpression mutants have an increased number of mesophyll cells between the vasculature tissue and show reductions in the size of the BS cells (S3 Table). Leaf M architecture greatly affects CO_2_ diffusion from the intercellular air space (IAS) to the chloroplast stroma, influencing photosynthetic activity [30,31], leaf hydrology and transpiration [32–34]. It remains to be established how variation in M architecture is related to the overexpression of *OsSAP16* and the effect this has on photosynthetic rate.

### *OsSAP16* regulates the expression of a broad range of stress response genes

Our results show that *OsSAP16* regulates the expression of a wide range of genes that function in regulating the response to stress (S6 Fig). The top ten most highly expressed genes, each with a 5–10 fold increase in expression, encode receptor like kinases (RLK) and abiotic stress response genes (S7D Fig). RLKs are implicated in a diverse range of poorly characterized signaling processes [35]. Increasing evidence suggests that these can play both a positive or negative role in stress response [35]. Recently, the leucine rich repeat RLK GUARD CELL HYDROGEN PEROXIDES-RESISTANT1 (GHR1) has been shown to interact by phosphorylation with the anion channel slow anion channel-associated1 (SLAC1) resulting in stomatal closing under drought stress [36]. While further studies are required to understand potential interactions between *OsSAP16*, RLKs and stomatal closure.

### Expression of *OsSAP16* is enhanced under drought conditions

Our results show that expression of *OsSAP16* is enhanced under drought conditions in wild type plants. In mutant plants expression decreased relative to plants grown under well watered conditions (Fig 6), although remained several fold higher than in the wild type. No significant differences in the response of the physiology of mutants to drought were found (Table 1).

Expression of *OsSAP1*6 has previously been reported to be upregulated by wounding, the application of the signaling molecules salicylic acid (SA) and H_2_O_2_ [37] and in response to cold, salt and dehydration stress [21]. *OsSAP16* is closely homologous to two SAP genes from *Arabidopsis*, which play important roles in tolerance to abiotic stresses. *AtSAP11* shows differential expression under abiotic stresses such as heavy metal and metalloids (As, Cd and Zn), high and low temperatures and salt [38]. Overexpression of the gene in transgenic plants has been shown to confer moderate tolerance to heavy metals and drought stress [38]. *AtSAP13* shows differential expression when plants are treated with H_2_O_2_ [39]. This suggests that SAP genes with close homology within diverse plant species play an important role in modulating the response of plants to both abiotic and biotic stress.

### Molecular functions of *OsSAP16* in stomatal movement

Stomatal opening is driven by the accumulation of K^+^ salts and/or sugars in the guard cells resulting in a decrease in water potential and uptake of water into the cell via osmosis [40]. This increases the guard cell volume leading to a widening of the stomatal aperture. An efflux of K^+^ leads to an increase in water potential and loss of water from the guard cell driving stomatal closure [41]. Stomatal aperture is adjusted in response to a multitude of environmental signals such as light [40], humidity [42], and CO_2_ [43]. Stomatal closure plays important role in the response of plant to both abiotic and biotic stresses [44,45], including drought in order to reduce the amount of water lost through transpiration [46]. As such there exists a conflict between high-yield characteristics and drought-tolerance traits [47–49].

Our data indicates that overexpression of *OsSAP16* may lead to a decrease in *g*_*s*_ through regulating the expression of genes encoding transporters, especially proteins in ABC transporter family. Three ABC transporter genes were significantly downregulated in the two mutants, namely LOC_Os12g32814 encoding a protein similar to ATPDR13, LOC_Os08g30770 encoding a protein identical to ATATH6, and LOC_Os12g13720 encoding a protein identical to ATPDR9. Regulation of stomatal movement by different types of ABC transporters has been widely studied, i.e. AtMRP5 [50], AtMRP4 [51], PDR-type ABC transporter [52], AtABCB14 [53], and AtABCG22 [54]. These ABC transporters affects plant stomatal movement and water transpiration via hormonal signaling like uptake of abscisic acid [51,52] or transporting organic anions like malate [50,53]. Moreover, one gene encoding OsCML28, one calmodulin-related calcium sensor protein, was significantly down regulated in the two mutants. Calcium and calmodulin were involved in the responses of stomata to abscisic acid mediated by heterotrimetic G protein and H_2_O_2_ [55–57]. The role of these candidate genes in regulating stomata movement deserves further demonstration.

Several transcription factors controlling stomatal movement have previously been characterized, with the majority of these functioning in the ABA signaling pathway [58]. The first TF to be identified were members of the MYB TF family in *Arabidopsis*. AtMYB60 is a positive regulator of stomatal opening [59], while AtMYB61 and AtMYB15 are both negative regulators of stomatal opening [60,61]. Members of the WRKY TF family (WRKY46, WRKY70 and WRKY54) have also been found to regulate stomatal aperture in *Arabidopsis* [62,63]. In rice, three TFs have been identified, SNAC1, DST and OsRZFP34 [64–66]. Overexpression of SNAC1 increased stomatal closure through regulating a large number of stress-related genes [64]. DST negatively regulates stomatal closure by modulating the expression of genes related with H_2_O_2_ homeostasis [65]. OsRZFP34 positively regulates stomatal closure by modulating the expression of genes implicated in Ca^2+^ sensing, K^+^ regulator, ABA response [66]. To the best of our knowledge *OsSAP*16 has not previously been shown to regulate stomatal conductance. However, expression of *OsSAP16* and the homologous gene *AtSAP13* in *Arabidopsis* has been shown to be induced by hydrogen peroxide [37,39], an important intermediate in ABA induced stomatal closure [67,68].

There is increasing evidence for crosstalk between abiotic and biotic signaling networks [69], together with a significant overlap in the signaling and regulatory components associated with the response to these stresses [70,71]. Recently the signaling molecules ABA, ROS, NO, OST1 kinase known to function in biotic stress induced stomatal closure have recently also been shown to be involved in pathogen/microbe-associated molecular patterns (MAMPs) induced stomatal closure [44,45,72,73]. The mode of action through which these gene expression changes or the reduced stomatal conductance phenotype is mediated by *OsSAP16* is not known, however, these results suggest a regulatory role for *OsSAP16* similar to that of *OsSAP1* [37,74].

Understanding the mechanisms of how plants sense and respond to biotic stress is an area of intense research interest in order to identify opportunities to improve tolerance at the molecular level. Here, we reveal new potential insights in the role of stress associated proteins in regulating stomatal conductance.

## Materials and Methods

### Plant materials

T-DNA activation tagging mutants were obtained from the Pohang University of Science and Technology [17,18]. This population uses four *35S* cauliflower mosaic virus (CaMV) promoters positioned near the border of the T-DNA to activate expression of genes located within 10 kb of the site of insertion in the *japonica* genetic background *Oryza sativa* cv. Dongjin (DJ). Two mutants were selected from this population, 3A-6000 and 3A-50378, hereafter refereed to as Ac1 and Ac2.

### Plant growth conditions

All measurements, except for those made on drought stressed plants, were made on plants grown in a greenhouse at the International Rice Research Institute (Los Baños, Philippines-14°9′53.58′′S 121°15′32.19′′E) during either the wet or dry season of 2013. Unless otherwise stated, the same generation of wild type and azygous plants were grown as the mutants. Dormancy was broken by incubation at 50°C for 5 days and seeds germinated on moist filter paper in the dark at 30°C. Seedlings were sown into soil sourced from the IRRI upland farm in 96-well trays and then transplanted into 0.5 L pots at the 2–3 leaf stage. Nutrients (0.09–0.01–0.09 g NPK kg^-1^) were incorporated as a basal dressing at the start of the experiment with 0.09 g N kg^-1^ soil added every 3 weeks. Plants were watered daily and insects were intensively controlled.

For measurements made on plants under drought stress, plants were grown in a greenhouse at IRRI from 4^th^ July to 2^nd^ August 2013. Seedlings of T_4_ generation Ac2 T_5_ DJ and Ac1 plants were sown directly into mylar tubes (50 x 5 cm) filled 10 cm from the top with dry fine-sieved soil from the IRRI upland farm. Two experimental treatments were imposed as previously described [75]. In the well watered (WW) treatment the soil in the tubes was soaked for 2 hours and left to drain overnight to achieve field capacity. One week after sowing, plants in this treatment were watered every other day in order to maintain a water content 1.2x field capacity. The amount of water added to each tube was recorded and used to calculate water uptake (mL) during the experiment (S3 Fig). The soil in the dry-down treatment (DD) was at 70% field capacity at the start of the experiment, no additional water was supplied to these plants. Water uptake was calculated as the difference in weight of each tube from the start to the end of the experiment. Ten plants per line were grown for each treatment.

### PCR screening for genotyping

DNA was extracted from homozygous T_3_ (Ac2) and T_4_ (Ac1) generation plants following the method from [76]. Genotyping was performed according to the protocol from [77] using three pairs of primers, two for the genomic DNA on both sides of the site of insertion and one for the border of the T-DNA (S1 Table). T-DNA insertion sites of the two mutants were determined based on the flanking sequences available from the RiceGE database (http://signal.salk.edu/cgi-bin/RiceGE).

### DNA blot analysis

Eight μg of genomic DNA was extracted and digested overnight at 37°C using the restriction endonuclease *ClaІ* (New England Biolabs Inc.). DNA was fractionated on a 0.8% agarose gel and the genomic DNA was transferred onto a nylon membrane (Amersham, GE Healthcare Life Sciences) by blotting overnight. DNA was cross-linked to the membrane under UV light and a *gus* gene fragment (S1 Table) used as a probe to hybridize the blot overnight at 50°C. After washing, CDP-star (Amersham, GE Healthcare Life Sciences) was applied evenly over the entire surface, the blot was incubated for 5 min at room temperature and placed in a cassette in contact with X-ray film (Amersham, GE Healthcare Life Sciences) and exposed for 90 min.

### RT-PCR

Leaves for RT-PCR were sampled from homozygous T_5_ generation Ac1 and T_4_ generation Ac2 plans two weeks after sowing. cDNA was synthesized using Transcriptor High Fidelity cDNA synthesis Kit (Roche Applied Science, USA). PCR analysis was performed using the synthesized cDNA with gene specific primers and primers for the 18S rRNA house keeping gene (S1 Table).

### Stomatal characterization

Epidermal impressions were taken from 5 penultimate leaves from homozygous T_5_ generation plants of Ac1, T_4_ generation plants of Ac2 and T_5_ of DJ. Finger nail polish was applied to the lower and upper epidermis of the middle portion of fully expanded leaves and allowed to dry for 10–20 min. A piece of clear cellophane tape was placed over the section of nail polish and carefully peeled from the leaf and the ‘impression’ was transferred to a microscope slide. Imprints were viewed under an Olympus BX51 compound microscope (Olympus, Japan) equipped with an Olympus DP71 camera (Olympus, Japan). Images were saved to a computer and analysed in ImageJ (http://imagej.nih.gov/ij/). Stomatal density (Sden), stomatal index (SI), the number of stomatal rows (SR), stomatal distance (SD), epidermal cell width (EPW), epidermal cell density (Eden) and epidermal cell height (EPH) were measured over an area of 0.25mm^2^ with a magnification of 200x. Where, SD is the distance between stomata and SI is the ratio of number of stomata per total number of epidermal cells. Stomatal width (SW) and stomatal height (SH) were measured on 25 randomly selected stomata at a magnification of 400x.

### Leaf anatomy

Leaf samples from homozygous T_5_ generation plants of Ac1, T_4_ generation of Ac2 and T_5_ generation plants of DJ were collected and immediately fixed in formaldehyde-acetic acid-alcohol (FAA volume ratios). Leaves were cut into thin transverse sections manually using a double-edged disposable razor blade on a rubber cutting mat [78]. The sections were dehydrated in 85% ethanol overnight and cleared with lactic acid. The clearing solution was then removed with a pipette and the sections were thoroughly washed several times with distilled water. Sections were mounted on a microscope slide and the orientation checked before being covered with a glass slide. Any thick or damaged sections were discarded; the leaf anatomical features listed in S2 Table were measured on 5–10 sections from one leaf from five plants per line.

### Leaf chlorophyll content

One leaf from eight different homozygous generation plants were pooled and ground into fine powder under liquid nitrogen; replicated three times. Chlorophyll was extracted from 0.2 g of material in 98% (v/v) acetone. The resulting extracts were centrifuged at 1600 g at 4°C for 1 min and the absorption at 645 nM and 663 nM measured in a spectrophotometer (Cary 60 UV-Vis, Agilent Technologies). The content of chlorophyll *a*, *b*, *a+b* and the ratio of chlorophyll *a* to *b* (*a/b*) was calculated as follows:

Chlorophyll a (mg/g fresh weight) = (12.7 A663) − (2.69 A645) / fresh weight × V

Chlorophyll b(mg/g fresh weight) = (22.9 A645) − (4.68 A663)/g fresh weight × V

Chls a+b (mg/g fresh weight) = ((20.08 A645) + (8.02A663))/ g fresh weight × V

where V equals 2 mL of extract.

### Photosynthesis measurements

Leaf gas-exchange measurements were made at IRRI (mean atmospheric pressure of 94.8 kPa) using a LI-6400XT portable photosynthesis system (LICOR Biosciences, Lincoln, NE, USA). Instantaneous leaf measurements of CO_2_ assimilation (*A*) were made at a photosynthetic photon flux density (PPFD) of 1500 μmol photons m^-2^ s^-1^ and a *C*_*a*_ of 400 μmol CO_2_ mol^-1^. Measurements were taken between 8 am and 12 pm on the seventh fully expanded leaf of 28–69 progenies per line.

Photosynthetic response curves to changing light intensity were acquired by increasing PPFD from 0 to 2000 μmol photons m^-2^ s^-1^ at a *C*_*a*_ of 400 μmol CO_2_ mol^-1^. The response of *A* to changing CO_2_ concentration (*A-C*_i_) was acquired by increasing *C*_*a*_ from 0 to 1500 μmol CO_2_ mol^-1^ at a PPFD of 1500 μmol photons m^-2^ s^-1^. Penultimate leaves on the main tiller were used for the measurement at heading stage. Measurements were made on one leaf from eight mutants and wild type plants. Leaves were acclimated for approximately 30 min before measurements were made under the following conditions, air temperature (T_air_) of 27°C, relative humidity of 60–70%, air flow rate of 400 μmol s^-1^ and a leaf-to-air vapor pressure deficit (VPD) of between 1.0–1.5 kPa.

Net CO_2_ assimilation rate (*A*, μmol CO_2_ m^-2^ s^-1^), stomatal conductance to water vapor (*g*_*s*,_ mol m^-2^ s^-1^) and leaf intercellular CO_2_ partial pressure (*C*_*i*_, μmol CO_2_ mol^-1^), ambient CO_2_ partial pressure (*C*_*a*_, μmol CO_2_ mol^-1^) and *C*_*i*_*/C*_*a*_ were extracted from the dataset. Intrinsic water use efficiency (iWUE) was calculated from the dataset as *A*/*g*_*s*_ at *C*_*a*_ of 400 μmol CO_2_ mol^-1^. The carboxylation efficiency (CE, μmol CO_2_ m^-2^ s^-1^) and CO_2_ compensation point (Г, μmol CO_2_ mol^-1^) were calculated from the initial slope (*Ci* < 50) of the *A-Ci* curves. Quantum yield (*φ*) was calculated from the initial slope (PPFD <200) of the light curves.

### Plant growth analysis, destructive harvesting and analysis of roots

Twenty days after the start of the drought experiment, measurements of plant height were made and then plants were destructively harvested. Leaves and sheaths were immediately dried to a constant weight in an oven at 70°C. Roots were cleaned and scanned at 600 dpi with a HP Scanjet 8200 and then dried to a constant weight. Root images were analyzed to measure maximum root depth, total root length, surface area, average root diameter, number of tips, forks and the percentage of lateral root using the WinRHIZO v. 2005 (Regent Instruments, Quebec, Canada). Water use efficiency (WUE) was calculated as the ratio of total dry weight (root and shoot) to water uptake during the experiment.

### RNA sequencing

Frozen leaf samples of T_5_ generation Ac1, T_4_ generation Ac2 and T_5_ generation DJ plants were ground into fine powder with a mortar and pestle. Five mL of TRIZOL^™^ (Dongsheng Biotech, Guangzhou) reagent was added and mixed gently at room temperature. One mL of chloroform:isoamyl alcohol (24:1) was added and the extract vortexed twice for 30 seconds at room temperature to dissolve the nucleo-protein complex. The solution was centrifuged at 14,000 rpm at 4°C for 10 min, the supernatant collected and mixed with 1 mL of chloroform:isoamyl alcohol (24:1). Centrifugation was repeated to obtain the supernatant, which was then mixed with 5 mL of isopropanol and 1 mL of 4 M NaCl to precipitate the RNA. The solution was incubated for 1 hour at -20°C, centrifuged at 14,000 rpm and 4°C for 30 min to obtain the RNA pellet. This was washed twice with 1 mL of 75% ethanol, dried over ice and autoclaved nanopure water added to dissolve RNA. The concentration of RNA was quantified using a NanoDrop 8000 (Thermo Scientific, Wilmington, USA) and the integrity was checked by running 1 μg of RNA on a 2% agarose gel at 130 volts for 1 hour. RNA was purified using RQ1 RNase-free DNase kit (Promega, USA) following the manufacturer’s instructions. The purified RNA was sent to Beijing Genomics Institute (BGI), Hong Kong for RNA-sequencing. The size-selected libraries were made for 200 bp cDNA fragments using an Illumina's standard RNA-seq library preparation protocol and sequenced 100 bp from both ends (100-bp paired-end) using HiSeq 2000 (Illumina, USA). Forty million reads, equivalent to 4 Giga bp clean bases were obtained for bioinformatics analysis

The paired-end RNA-seq data obtained from the nine samples (three replications for Ac1, Ac2 and DJ) were first assessed for the overall read quality (per base quality, per sequence quality scores, per sequence GC content) using the FASTQC tool (http://www.bioinformatics.babraham.ac.uk/projects/fastqc/). All samples contained good-quality sequences with an average PHRED quality per read of 37–38. The average GC content of overall reads in each sample varied between 48 to 50 percent.

Read error correction was performed for one iteration using the error correction module of ALLPATHS-LG tool [79]. The error-corrected reads from each library were then used for gene expression quantification with the RSEM tool [80]. RSEM reported quantity of expression level of each gene in three formats: expected counts, TPM (transcript per million) and FPKM (fragment per kb of effective length per million).

The expected counts were collected and converted to an expression matrix with gene ids in rows and samples in columns. Three R-based tools were chosen for DE analysis, i.e. baySeq [81], DESeq [82] and edger [83]. The DE analyses using those three programs were done in a pair wise manner with three replications of the samples, i.e. DJ (3 reps) vs Ac1 (3 reps), and DJ (3 reps) vs Ac2 (3 reps). Differentially expressed genes were identified based on adjusted *p*-values or false discovery rate (FDR). Overlapping genes from the three methods with adjusted *p-*value less than 0.1 were selected for enrichment analysis.

The enrichment analysis was performed in the AgriGO website (http://bioinfo.cau.edu.cn/agriGO/analysis.php), the statistical test method used was Fisher at a significant level of 0.05. The gene annotation was based on Osa_MSU_v7 using Mapman 3.5.1R2.

### Statistical analysis

Statistical analyses were performed in Statistix 8 using analysis of variance (ANOVA; with a *p*-value of 0.05). Statistically significant differences were determined using a least significant difference (LSD) test.

## Supporting Information

S1 Fig Analysis of the *OsSAP16* gene and the protein encoded by the gene. A, gene structure of *OsSAP16*. B, conserved AN1 and C2H2 zinc finger (ZnF) domains in the protein encoded by *OsSAP16*. C, phylogenetic tree of the stress associated protein (SAP) family in *Arabidopsis* (At), maize (Zm) and rice (Os). Amino acid sequences from NCBI were used for construction of the tree.
(TIF)

S2 Fig Relative expression profile of *OsSAP16* in different cell types and plant tissues of rice. Data is captured from ROAD (http://www.ricearray.org/) based on the study by Jiao *et al*. (2009, *Nat*. *Genet*. **41,** 258–63).
(TIF)

S3 Fig Soil water content of DJ (Dongjin), Ac1 and Ac2 under two water treatments. (TIF)

S4 Fig A, Carboxylation efficiency (CE), B, CO_2_ compensation point (*Γ*), and C, quantum yield (*φ*) of Dongjin (DJ), an azygous mutant line (Ac2_azygous) and two *OsSAP16* overexpression mutants (Ac1 and Ac2). Values are the average ± SE of one leaf from eight plants for each line. Different lower case letters indicate a statistically significant difference at the 0.05 level.
(TIF)

S5 Fig Chlorophyll content in the penultimate leaves of DJ (Dongjin) and two *OsSAP16* overexpression mutants (Ac1 and Ac2). Values are the average ± SE of 3 replicates (each replicate is one leaf pooled from 8 plants).
(TIF)

S6 Fig Venn diagrams of the number of significantly down- and up-regulated genes in *OsSAP16* overexpression mutants Ac1 and Ac2 compared to the wild type Dongjin (DJ). A, comparison of the number of genes identified using three different differential gene expression analysis methods (baySeq, DESeq and edgeR). B, number of unique and overlapping genes identified in both mutants using all 3 methods.
(TIF)

S7 Fig Pathway analysis of the significantly down- and up-regulated genes in two *OsSAP16* overexpression mutants. A, genes involved in stress response. B, genes involved in primary metabolism. C, genes involved in secondary metabolism. D, genes encoding receptor like kinases. The analysis was performed in Mapman3.5.1R2. Red and blue colors indicate log2 fold changes of up- and down-regulated genes, respectively.
(TIF)

S8 Fig GO enrichment analysis of the significantly down- and up- regulated genes in two *OsSAP16* overexpression mutants. A: biological process of down- regulated genes; B: biological process of up- regulated genes; C: Cellular components of up- regulated genes; D: Molecular function of up- regulated genes. No significant cellular components or molecular functions were identified for down- regulated genes. The analysis was performed in AgriGO with singular enrichment analysis (SEA) tool. Statistical test method was Fischer with no multi-test adjustment, and the significance level was at 0.05.
(TIF)

S1 Table Gene specific primers used for genotyping, RT-PCR and southern blot. (DOCX)

S2 Table Characterization of leaf anatomy in Dongjin (DJ) and two *OsSAP16* overexpression mutants (Ac1 and Ac2). (DOCX)

S3 Table Stomatal characterization of the abaxial and adaxial leaf surface of Dongjin (DJ) and two *OsSAP16* overexpression mutants (Ac1 and Ac2). (DOCX)

S4 Table Characterization of root traits of Dongjin (DJ) and two *OsSAP16* overexpression mutants (Ac1 and Ac2) grown under two water treatments. (DOCX)

S5 Table Significantly down- and up-regulated genes with known functions in two OsSAP16 overexpression mutants (Ac1 and Ac2) compared to Dongjin (DJ). Inf indicates there was no expression of the gene in the mutant or DJ, respectively.
(DOCX)
