# Supplementary figures and images for: Overexpression of OsSAP16 Regulates Photosynthesis and the Expression of a Broad Range of Stress Response Genes in Rice (Oryza sativa L.)
Source: PLoS One. 2016 Jun 15;11(6):e0157244. doi: 10.1371/journal.pone.0157244 (PMC4909303; doi:10.1371/journal.pone.0157244)

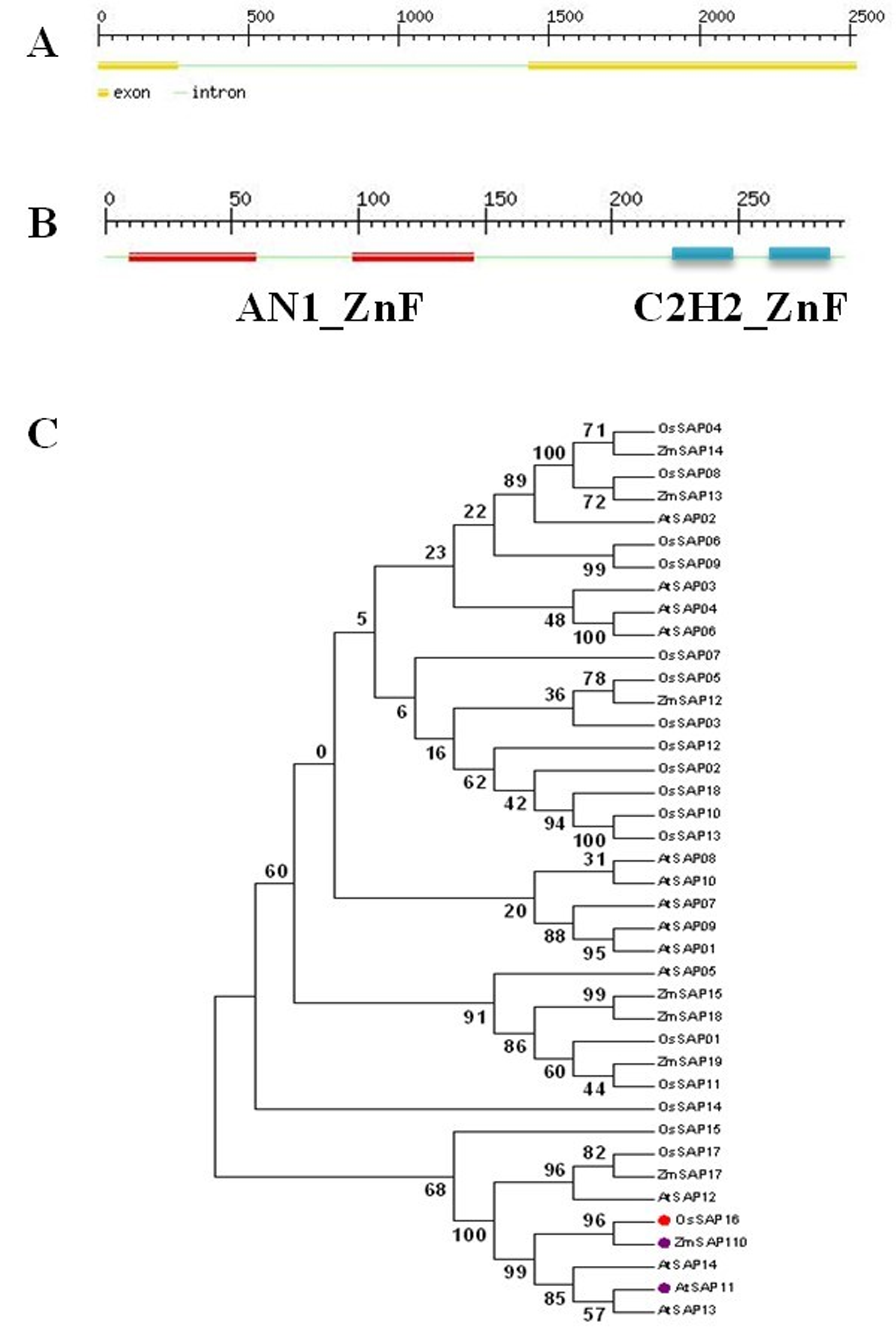

Supplement: S1 Fig — A, gene structure of OsSAP16. B, conserved AN1 and C2H2 zinc finger (ZnF) domains in the protein encoded by OsSAP16. C, phylogenetic tree of the stress associated protein (SAP) family in Arabidopsis (At), maize (Zm) and rice (Os). Amino acid sequences from NCBI were used for construction of the tree. (TIF) [file pone.0157244.s001.tif]

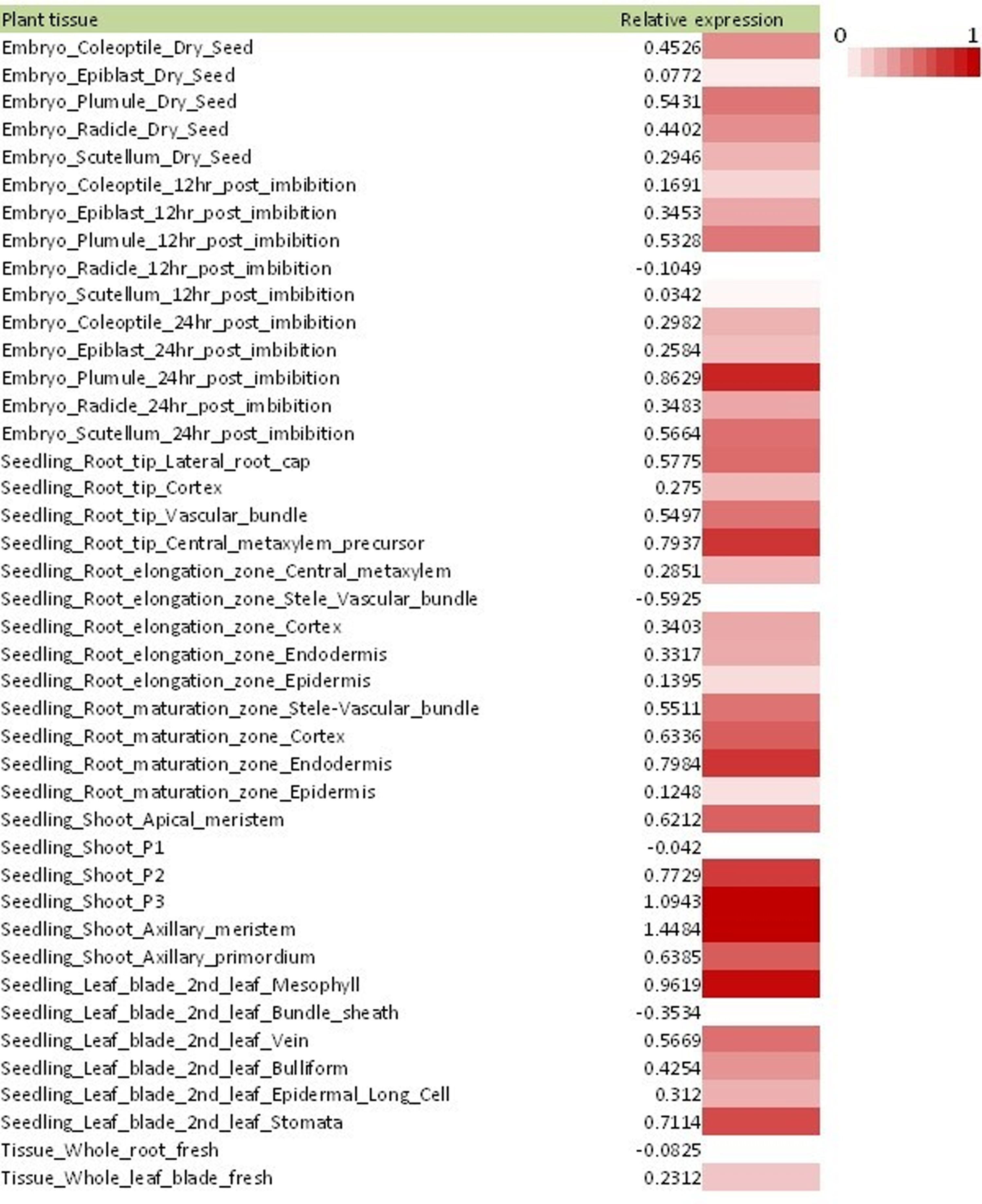

Supplement: S2 Fig — Data is captured from ROAD (http://www.ricearray.org/) based on the study by Jiao et al. (2009, Nat. Genet. 41, 258–63). (TIF) [file pone.0157244.s002.tif]

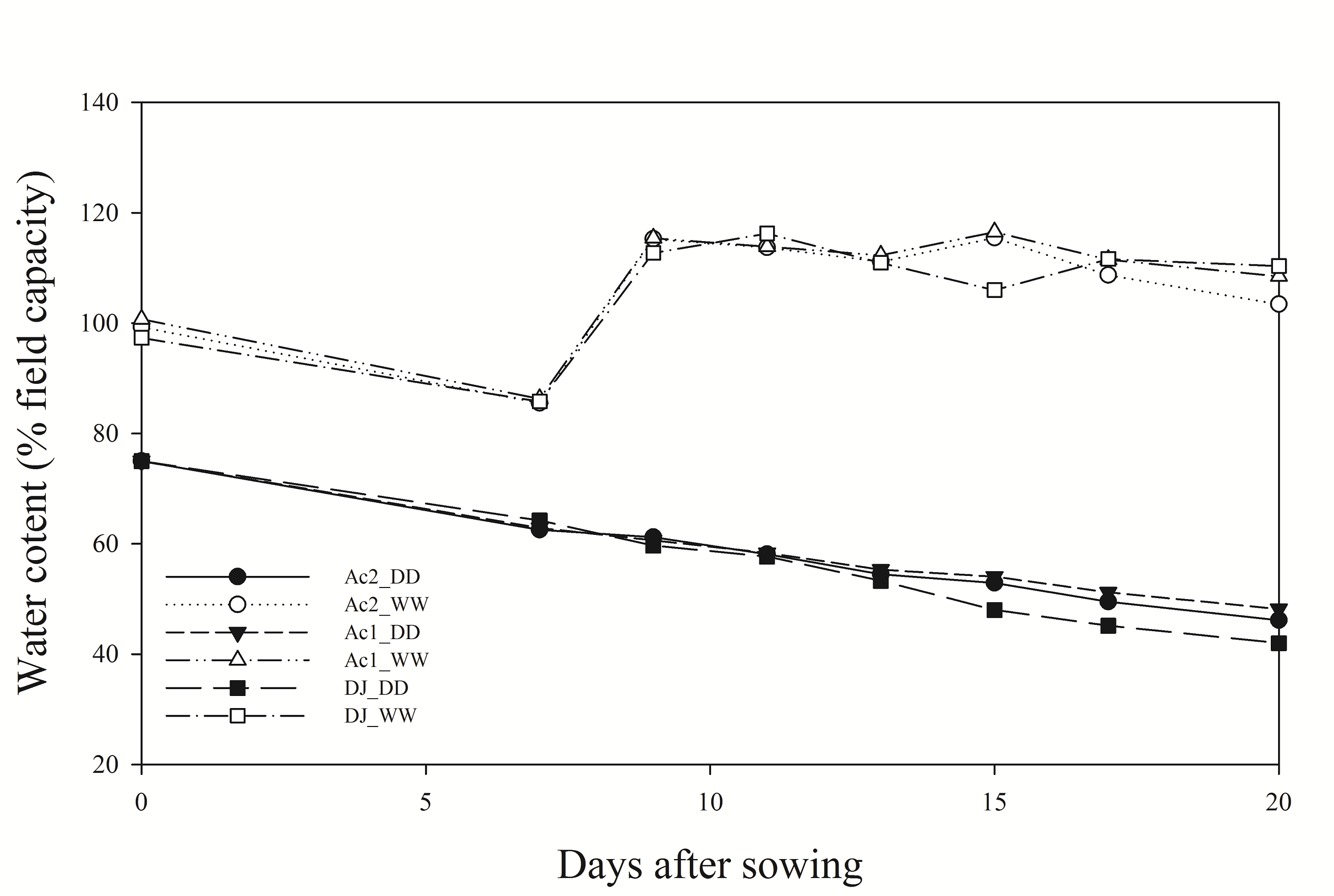

Supplement: S3 Fig — (TIF) [file pone.0157244.s003.tif]

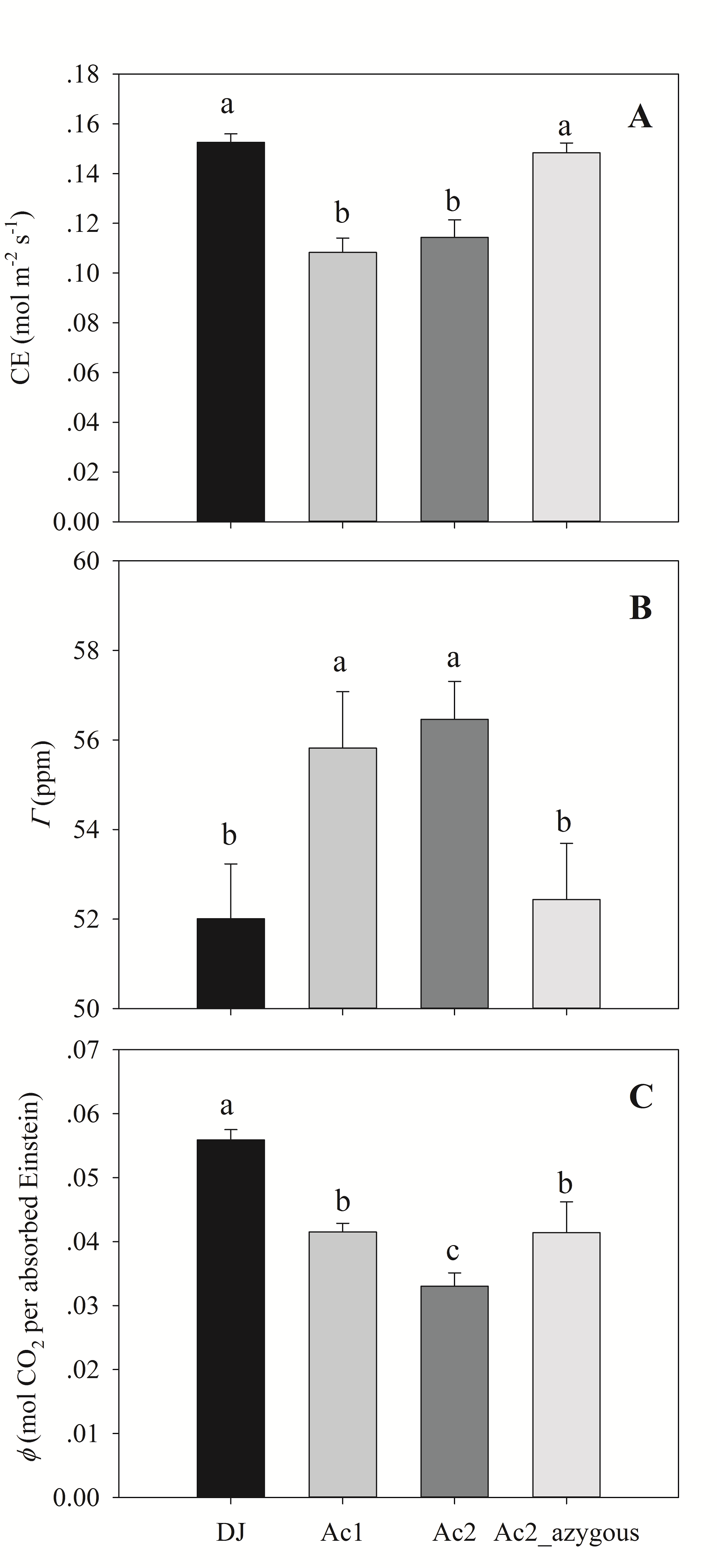

Supplement: S4 Fig — Values are the average ± SE of one leaf from eight plants for each line. Different lower case letters indicate a statistically significant difference at the 0.05 level. (TIF) [file pone.0157244.s004.tif]

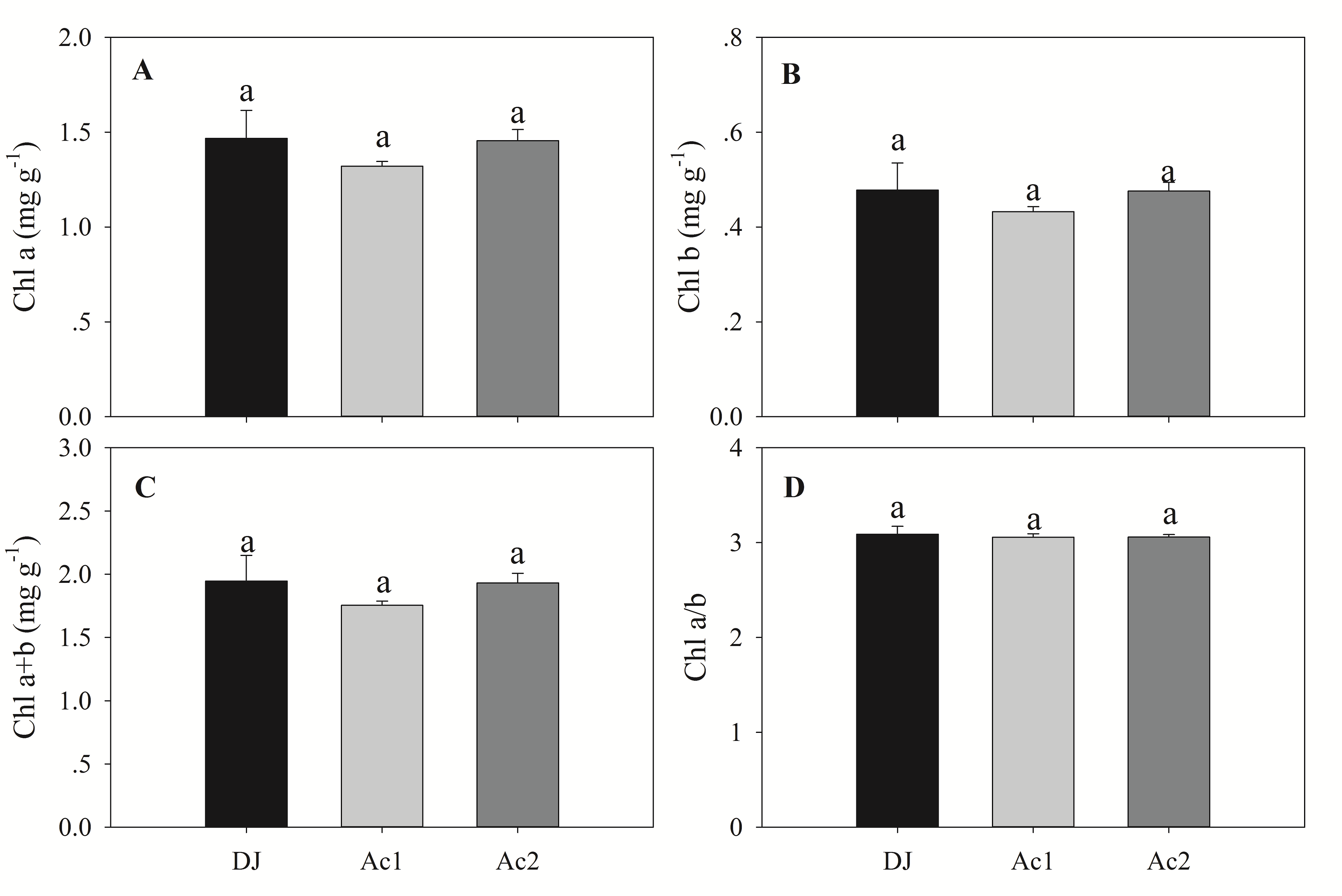

Supplement: S5 Fig — Values are the average ± SE of 3 replicates (each replicate is one leaf pooled from 8 plants). (TIF) [file pone.0157244.s005.tif]

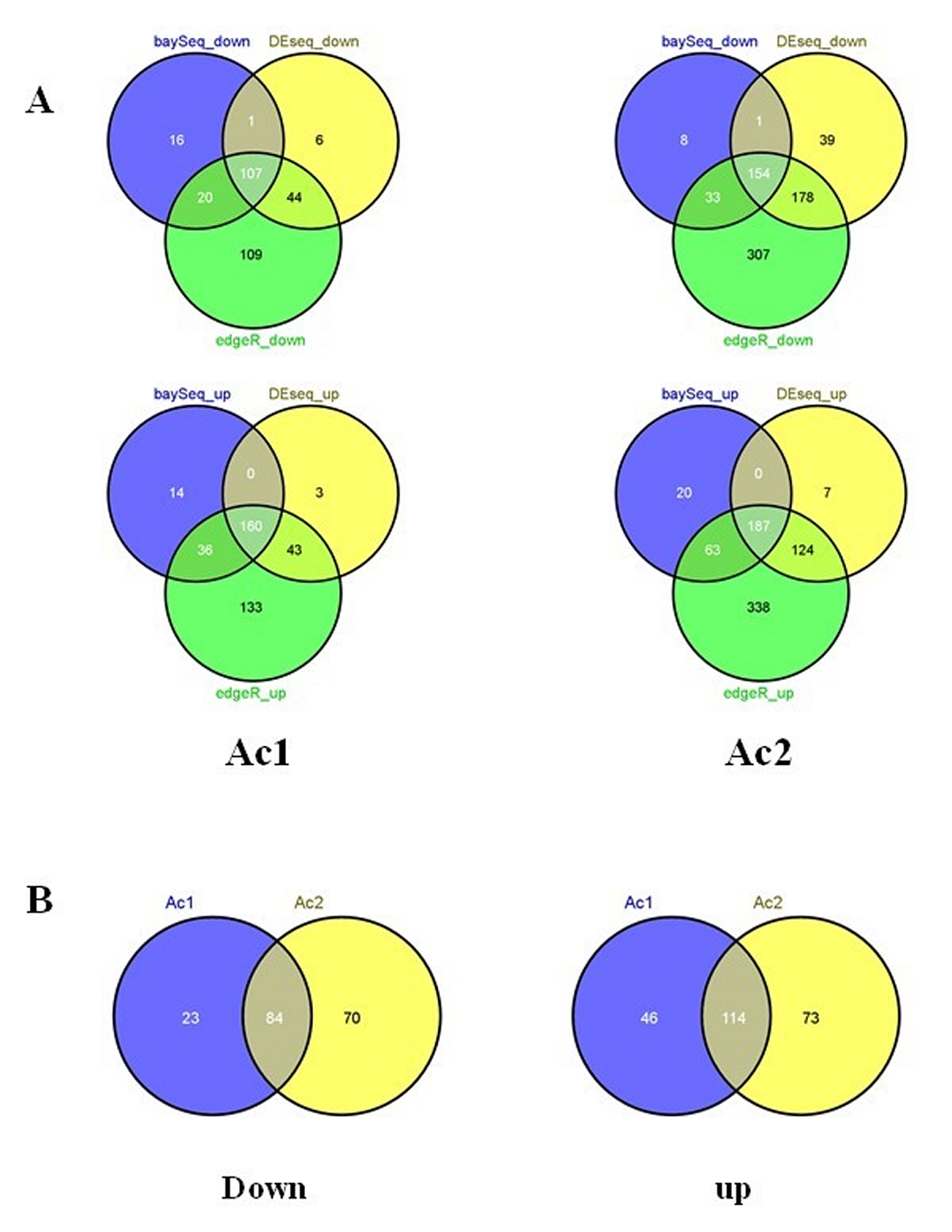

Supplement: S6 Fig — A, comparison of the number of genes identified using three different differential gene expression analysis methods (baySeq, DESeq and edgeR). B, number of unique and overlapping genes identified in both mutants using all 3 methods. (TIF) [file pone.0157244.s006.tif]

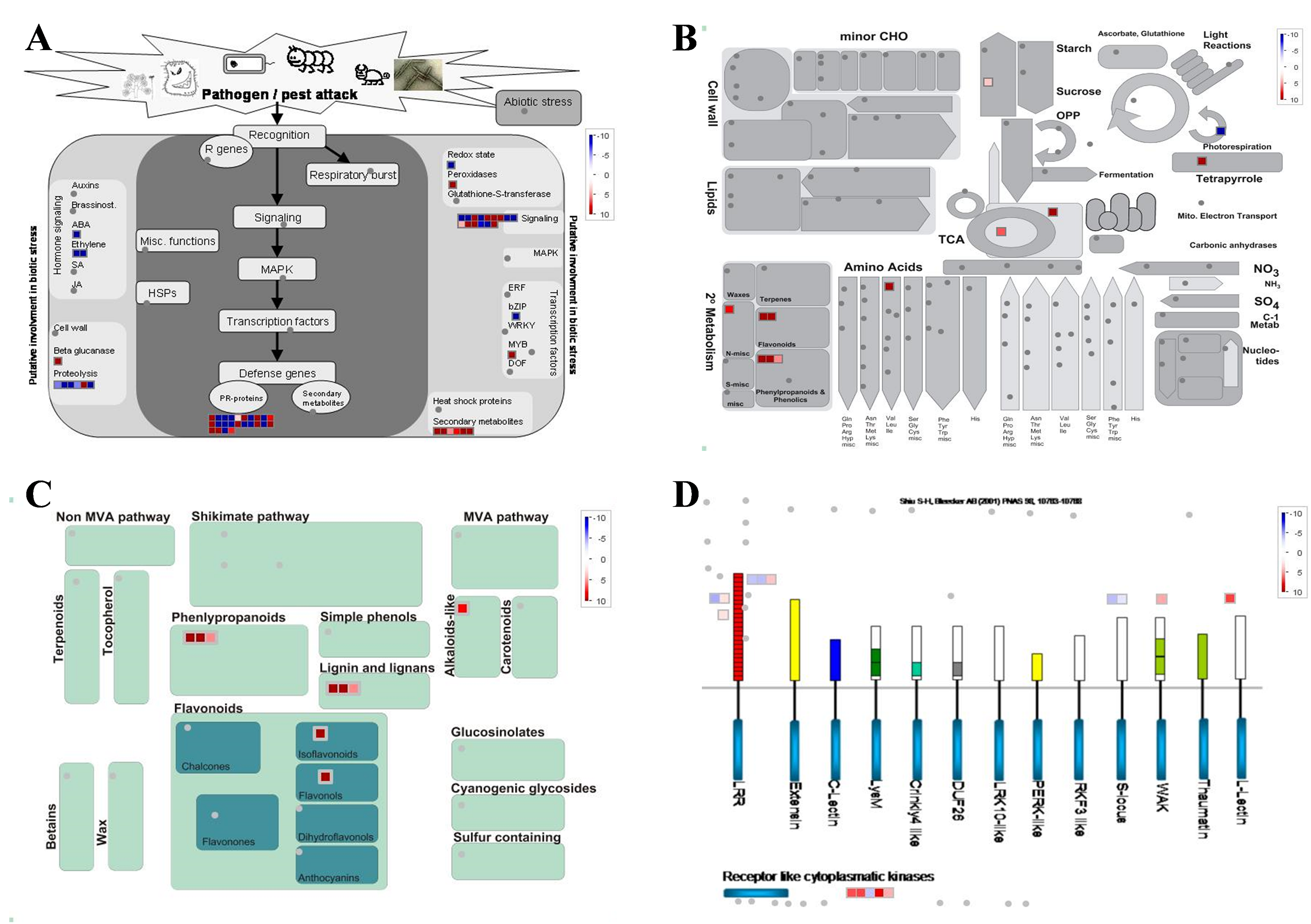

Supplement: S7 Fig — A, genes involved in stress response. B, genes involved in primary metabolism. C, genes involved in secondary metabolism. D, genes encoding receptor like kinases. The analysis was performed in Mapman3.5.1R2. Red and blue colors indicate log2 fold changes of up- and down-regulated genes, respectively. (TIF) [file pone.0157244.s007.tif]

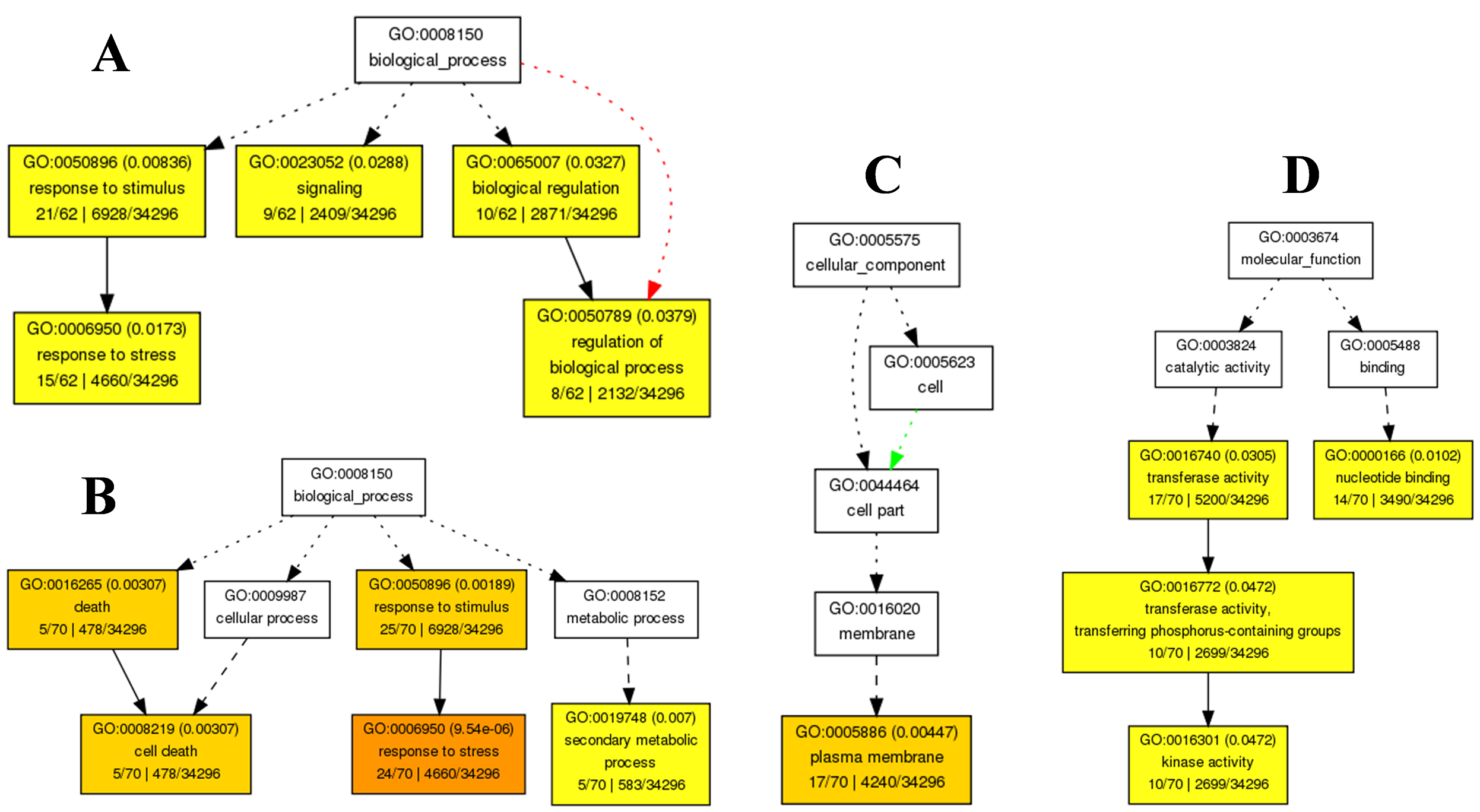

Supplement: S8 Fig — A: biological process of down- regulated genes; B: biological process of up- regulated genes; C: Cellular components of up- regulated genes; D: Molecular function of up- regulated genes. No significant cellular components or molecular functions were identified for down- regulated genes. The analysis was performed in AgriGO with singular enrichment analysis (SEA) tool. Statistical test method was Fischer with no multi-test adjustment, and the significance level was at 0.05. (TIF) [file pone.0157244.s008.tif]
