# Supplementary material for: Overexpression of OsSAP16 Regulates Photosynthesis and the Expression of a Broad Range of Stress Response Genes in Rice (Oryza sativa L.)
Source: PLoS One. 2016 Jun 15;11(6):e0157244. doi: 10.1371/journal.pone.0157244 (PMC4909303; doi:10.1371/journal.pone.0157244)
Supplement: S1 Table — (DOCX) [file pone.0157244.s009.docx]

S1 Table. Gene specific primers used for genotyping, RT-PCR and southern blot.

| Set | Name | Primer | Primer Sequence (5' to 3') |
| --- | --- | --- | --- |
| Genotyping |  |  |  |
|  | Ac1 | Forward | TTTCAATTGAGCATTGGATCC |
|  |  | Reverse | GTGAGGCAATGAACCAAATTG |
|  | Ac2 | Forward | AAAACCTGCGCTAAGCAAAAG |
|  |  | Reverse | CCTGCACAATTCCGTTTTCTA |
|  | pGA_2715 | Left border | ATCTTGAACGATAGCCTTTCCTTTATCG |
| RT-PCR |  |  |  |
|  | *OsSAP16* | Forward | ATTGCGAAAAGTCCCATCAG |
|  |  | Reverse | TCTCAACATGCTCCACAAGC |
|  | LOC_Os07g38230 | Forward | CGGTGCTCCACCAGTTGTAT |
|  |  | Reverse | GTGTTTGTGTCGTCCGTTGG |
|  | 18S rRNA | Forward | ATGGTGGTGACGGGTGACG |
|  |  | Reverse | CAGACACTAAAGCGCCCGGTA |
| Southern blot |  |  |  |
|  | *gus* | Forward | GGCACAGCACATCAAAGAGA |
|  |  | Reverse | CCCTTACGCTGAAGAGATGC |
