# Supplementary material for: Overexpression of OsSAP16 Regulates Photosynthesis and the Expression of a Broad Range of Stress Response Genes in Rice (Oryza sativa L.)
Source: PLoS One. 2016 Jun 15;11(6):e0157244. doi: 10.1371/journal.pone.0157244 (PMC4909303; doi:10.1371/journal.pone.0157244)
Supplement: S2 Table — (DOCX) [file pone.0157244.s010.docx]

S2 Table. Characterization of leaf anatomy in Dongjin (DJ) and two *OsSAP16* overexpression mutants (Ac1 and Ac2).

|  |  | Line | | | | |
| --- | --- | --- | --- | --- | --- | --- |
| Parameter |  | DJ |  | Ac1 |  | Ac2 |
| Mesophyll Cell Number (MCNO) |  | 6.9±0.1^b^ |  | 7.4±0.14^a^ |  | 7.3±0.16^ab^ |
| Mesophyll Width (MCWD) | μm | 26.7±0.57^a^ |  | 26.1±1^a^ |  | 25.4±0.69^a^ |
| Mesophyll Cell Total Area (MCTA) | μm^2^ | 12068±300^a^ |  | 13000±1086^a^ |  | 11901±657^a^ |
| Small Vein Width (SVWD) | μm | 49.2±1.11^a^ |  | 46.2±1.81^ab^ |  | 44.2±1.47^b^ |
| Small Vein Height (SVHT) | μm | 51.7±1.63^a^ |  | 50.1±2.13^a^ |  | 52.6±2^a^ |
| Small Vein-Leaf Thickness (SVLFTH) | μm | 94.4±1.85^a^ |  | 99±2.86^a^ |  | 94.6±3.37^a^ |
| Interveinal Distance-Small Vein (SVIVD) | μm | 220±5.74^a^ |  | 228±7.48^a^ |  | 219±5.58^a^ |
| Bundle Sheath Cell Number-Small Vein (BSCNSV) |  | 10.3±0.22^a^ |  | 10.43±0.28^a^ |  | 9.83±0.34^a^ |
| Bundle Sheath Cell Area-Small Vein (BSCASV) | μm^2^ | 98.2±8.65^a^ |  | 76.3±7.72^ab^ |  | 71.1±4.93^b^ |
| Large Vein Width (LVWD) | μm^2^ | 133±5.25^a^ |  | 132±4.82^a^ |  | 127±4.53^a^ |
| Large Vein Height (LVHT) | μm | 131±6.17^a^ |  | 125±6.59^a^ |  | 124±3.81^a^ |
| Large Vein-Leaf Thickness (LVLFTH) | μm | 215±20^a^ |  | 202±5.6^a^ |  | 218±11^a^ |
| Interveinal Distance-Large Vein (LVIVD) | μm | 255±8.24^a^ |  | 261±6.37^a^ |  | 255±9.88^a^ |
| Bundle Sheath Cell Number-Large Vein (BSCNLV) |  | 18.4±0.4^a^ |  | 18.6±0.4^a^ |  | 18.1±0.72^a^ |
| Bundle Sheath Cell Area-Large Vein (BSCALV) | μm^2^ | 223±25.8^a^ |  | 178±18.5^ab^ |  | 146±3.9^b^ |

Data shown here are average ± SE of five leaves from five plants per line. Different letters between the three lines for each parameter indicate statistically significant difference at the 0.05 level.
