# Supplementary material for: Overexpression of OsSAP16 Regulates Photosynthesis and the Expression of a Broad Range of Stress Response Genes in Rice (Oryza sativa L.)
Source: PLoS One. 2016 Jun 15;11(6):e0157244. doi: 10.1371/journal.pone.0157244 (PMC4909303; doi:10.1371/journal.pone.0157244)
Supplement: S3 Table — (DOCX) [file pone.0157244.s011.docx]

S3 Table. Stomatal characterization of the abaxial and adaxial leaf surface of Dongjin (DJ) and two *OsSAP16* overexpression mutants (Ac1 and Ac2).

| Line | Sden | | SI | | | SR | | | SW (µm) | | | SH (µm) | | | SD (µm) | | | Eden | | EPW (µm) | | | EPH (µm) | |  |
| --- | --- | --- | --- | --- | --- | --- | --- | --- | --- | --- | --- | --- | --- | --- | --- | --- | --- | --- | --- | --- | --- | --- | --- | --- | --- |
| *Abaxial* |  |  |  |  |  | |  |  | |  |  | |  |  | |  |  | |  | |  |  | |  |  |
| DJ | 75±6.36^a^ |  |  | 23.3±0.69^b^ |  | | 1.69±0.10^a^ |  | | 23.7±0.94^a^ |  | | 16.1±0.60^a^ |  | | 23.7±1.10^a^ |  | | 246±13.35^a^ | | 54.5±2.69^a^ |  | | 16.4±0.66^a^ |  |
| Ac1 | 90±7.27^a^ |  |  | 26.6±0.93^a^ |  | | 1.92±0.06^a^ |  | | 22.2±1.20^a^ |  | | 15.8±0.37^a^ |  | | 24.2±1.39^a^ |  | | 245±11.15^a^ | | 58.3±3.09^a^ |  | | 14.2±0.39^b^ |  |
| Ac2 | 74±3.90^a^ |  |  | 25.8±0.86^ab^ |  | | 1.78±0.07^a^ |  | | 23.1±0.78^a^ |  | | 16.0±0.32^a^ |  | | 24.9±1.24^a^ |  | | 214±15.11^a^ | | 61.7±2.10^a^ |  | | 14.8±0.61^ab^ |  |
| *Adaxial* |  |  |  |  |  | |  |  | |  |  | |  |  | |  |  | |  | |  |  | |  |  |
| DJ | 65±3.04^a^ |  |  | 22.1±0.71^a^ |  | | 1.35±0.04^a^ |  | | 23.7±1.08^a^ |  | | 15.9±0.33^a^ |  | | 19.2±0.50^a^ |  | | 231±12.61^a^ | | 53.0±3.39^a^ |  | | 15.7±0.60^a^ |  |
| Ac1 | 69±7.07^a^ |  |  | 24.1±1.02^a^ |  | | 1.52±0.13^a^ |  | | 23.2±1.39^a^ |  | | 15.6±0.28^a^ |  | | 22.8±2.08^a^ |  | | 216±11.52^a^ | | 56.7±2.09^a^ |  | | 13.2±0.21^b^ |  |
| Ac2 | 57±3.65^a^ |  |  | 23.3±0.49^ab^ |  | | 1.23±0.07^a^ |  | | 24.2±1.00^a^ |  | | 15.6±0.35^a^ |  | | 24.6±1.02^a^ |  | | 191±12.79^a^ | | 58.3±3.12^a^ |  | | 13.4±0.46^ab^ |  |

Sden: stomatal density, Eden: epidermal cell density, SI: stomatal index, SR: number of stomatal rows, SW: stomatal width, SH: stomatal height, SD: distance between stomata, EPW: epidermal cell width, EPH: epidermal cell height.

Values are the average ± SE of five penultimate leaves per line.

Different letters between the three lines for each leaf surface indicate statistically significant differences at the 0.05 level.
