# Supplementary material for: Overexpression of OsSAP16 Regulates Photosynthesis and the Expression of a Broad Range of Stress Response Genes in Rice (Oryza sativa L.)
Source: PLoS One. 2016 Jun 15;11(6):e0157244. doi: 10.1371/journal.pone.0157244 (PMC4909303; doi:10.1371/journal.pone.0157244)
Supplement: S4 Table — (DOCX) [file pone.0157244.s012.docx]

S4 Table. Characterization of root traits of Dongjin (DJ) and two *OsSAP16* overexpression mutants (Ac1 and Ac2) grown under two water treatments.

| Treatment | Line | Maximum root depth (cm) | Total Root  Length  (cm) | Surface  Area  (cm^2^) | Average  Diameter  (mm) | Number  of tips | Number  of forks | Percentage  of lateral roots (%) |
| --- | --- | --- | --- | --- | --- | --- | --- | --- |
| DD |  |  |  |  |  |  |  |  |
|  | DJ | 39.8±1.45^a^ | 1519±128^a^ | 79.6±6.3^a^ | 0.168±0.0049^a^ | 10057±1055^a^ | 10714±1160^a^ | 76.1±1.02^a^ |
|  | Ac1 | 24.7±0.85^b^ | 842±107^b^ | 44.3±5.3^b^ | 0.168±0.0064^a^ | 4885±657^c^ | 5842±859^b^ | 76.7±1.53^a^ |
|  | Ac2 | 25.7±1.24^b^ | 1262±135^a^ | 68.5±6.8^a^ | 0.173±0.0029^a^ | 7864±804^b^ | 9259±980^a^ | 74.2±0.69^a^ |
| WW |  |  |  |  |  |  |  |  |
|  |  |  |  |  |  |  |  |  |
|  | DJ | 33.0±1.71^a^ | 1670±155^a^ | 88.8±6.6^a^ | 0.177±0.0035^a^ | 9885±872^a^ | 9752±999^a^ | 74.3±0.88^a^ |
|  | Ac1 | 18.5±0.75^b^ | 910±52^b^ | 51.0±2.1^b^ | 0.180±0.0085^a^ | 4749±384^c^ | 4634±385^b^ | 72.1±1.8^a^ |
|  | Ac2 | 21.8±0.84^b^ | 1342±110^a^ | 77.1±6^a^ | 0.186±0.0042^a^ | 6826±413^b^ | 6781±633^b^ | 72.3±0.7^a^ |
| AOV |  |  |  |  |  |  |  |  |
|  | Treatment | *** | ns | ns | * | ns | * | ** |
|  | Line | *** | *** | *** | ns | *** | *** | ns |
|  | Treatment  *Line | ns | ns | ns | ns | ns | ns | ns |

WW: well watered treatment, DD: dry down treatment, ANOVA: Analysis of Variance.

Values are the average ± SE of five plants for each line.

Different letters between the lines in each treatment indicate significant differences at the 0.05 level. Asterisks represent the significance for differences amongst lines, water treatments and lines×water treatment. *: p<0.05, **: p<0.01, ***: p<0.001, ns=not significant.
