# Supplementary material for: Overexpression of OsSAP16 Regulates Photosynthesis and the Expression of a Broad Range of Stress Response Genes in Rice (Oryza sativa L.)
Source: PLoS One. 2016 Jun 15;11(6):e0157244. doi: 10.1371/journal.pone.0157244 (PMC4909303; doi:10.1371/journal.pone.0157244)
Supplement: S5 Table — Inf indicates there was no expression of the gene in the mutant or DJ, respectively. (DOCX) [file pone.0157244.s013.docx]

S5 Table. Significantly down- and up-regulated genes with known functions in two *OsSAP16* overexpression mutants (Ac1 and Ac2) compared to Dongjin (DJ). Inf indicates there was no expression of the gene in the mutant or DJ, respectively.

|  |  | **log2FoldChange** | |  |
| --- | --- | --- | --- | --- |
| **#** | **Gene ID** | **Ac1** | **Ac2** | **DESCRIPTION** |
| 1 | LOC_Os01g08520.1 | -2.45 | -2.43 | DUF581 domain containing protein, expressed |
| 2 | LOC_Os01g11940.1 | -3.21 | -1.87 | Phosphatidylethanolamine-binding protein, expressed' |
| 3 | LOC_Os02g34490.1 | -3.84 | -3.97 | Leucine Rich Repeat family protein, expressed |
| 4 | LOC_Os02g49510.1 | -4.65 | -3.86 | amino acid transporter, putative, expressed |
| 5 | LOC_Os02g50330.1 | -1.78 | -1.52 | RNA-dependent RNA polymerase, putative, expressed |
| 6 | LOC_Os02g57860.1 | Inf | Inf | 'protein\|OsFBX71 - F-box domain containing protein, expressed' |
| 7 | LOC_Os02g58270.1 | -2.32 | -2.41 | \|metallo-beta-lactamase family protein, putative, expressed |
| 8 | LOC_Os04g01320.1 | -2.64 | -2.52 | serine/threonine-protein kinase receptor precursor, putative, expressed |
| 9 | LOC_Os04g01330.1 | -9.63 | Inf | 'protein\|expressed protein' |
| 10 | LOC_Os04g01470.1 | -2.19 | -2.52 | O-methyltransferase, putative, expressed |
| 11 | LOC_Os04g25540.1 | Inf | -5.68 | ribosomal protein L27, putative, expressed |
| 12 | LOC_Os04g42120.1 | Inf | -4.57 | N-acetyltransferase ESCO2, putative, expressed |
| 13 | LOC_Os07g08270.1 | -7.59 | Inf | expressed protein |
| 14 | LOC_Os07g16364.1 | -2.4 | -2.43 | 'protein\|expressed protein' |
| 15 | LOC_Os07g16600.1 | Inf | Inf | expressed protein |
| 16 | LOC_Os07g30250.1 | -1.96 | -2.03 | RFT1, putative, expressed |
| 17 | LOC_Os07g30280.1 | -3.51 | -3.14 |  |
| 18 | LOC_Os07g31190.1 | -4.92 | -5.88 | OsWAK71 - OsWAK receptor-like cytoplasmic kinase OsWAK-RLCK, expressed |
| 19 | LOC_Os08g03002.1 | Inf | -3.3 | lectin-like receptor kinase 1, putative, expressed |
| 20 | LOC_Os08g10290.1 | -4.38 | -4.09 | SHR5-receptor-like kinase, putative, expressed |
| 21 | LOC_Os08g10300.1 | -3.5 | -3.86 | SHR5-receptor-like kinase, putative, expressed |
| 22 | LOC_Os08g10440.1 | -3.78 | -3.5 | NBS-LRR disease resistance protein, putative, expressed |
| 23 | LOC_Os08g10730.1 | -4.96 | -3.85 | expressed protein |
| 24 | LOC_Os08g14195.1 | -10.35 | -8.86 |  |
| 25 | LOC_Os08g15149.1 | -1.91 | -1.63 | oxidoreductase, 2OG-Fe oxygenase family protein, expressed |
| 26 | LOC_Os08g17500.1 | -2.47 | -2.65 | cinnamoyl-CoA reductase, putative, expressed |
| 27 | LOC_Os08g18000.1 | -6.12 | -4.22 |  |
| 28 | LOC_Os08g30770.1 | -2.4 | -3.13 | ABC transporter, ATP-binding protein, putative, expressed |
| 29 | LOC_Os08g31000.1 | -2.38 | -2.37 | expressed protein |
| 30 | LOC_Os10g04570.1 | -3.65 | -4.53 | 'protein\|expressed protein' |
| 31 | LOC_Os10g04860.1 | Inf | -9.92 | AAO3 (Abscisic ALDEHYDE OXIDASE 3)aldehyde oxidase, putative, expressed' |
| 32 | LOC_Os11g03970.2 | -8.28 | -8.05 | CAMK_KIN1/SNF1/Nim1_like.5 - CAMK includes calcium/calmodulin depedent protein kinases, expressed' |
| 33 | LOC_Os11g27329.1 | -3.29 | -3.53 | \|OsSCP62 - Putative Serine Carboxypeptidase homologue, expressed |
| 34 | LOC_Os11g30790.1 | -1.72 | -1.71 | 'protein\|expressed protein' |
| 35 | LOC_Os11g32780.1 | Inf | Inf | 'protein\|expressed protein' |
| 36 | LOC_Os11g32810.1 | -10.38 | -10.47 | 'protein\|Leucine Rich Repeat family protein, expressed' |
| 37 | LOC_Os11g33970.1 | Inf | Inf | plant protein of unknown function domain containing protein, expressed |
| 38 | LOC_Os11g34270.1 | -3.5 | -3.7 | ubiquitin carboxyl-terminal hydrolase domain containing protein, expressed |
| 39 | LOC_Os11g43490.1 | Inf | Inf |  |
| 40 | LOC_Os11g44990.1 | Inf | -6.62 | NB-ARC domain containing protein, expressed |
| 41 | LOC_Os11g45090.1 | -5.1 | -4.98 | NB-ARC domain containing protein, expressed |
| 42 | LOC_Os11g45130.1 | -3.26 | -6.72 | pollen signalling protein with adenylyl cyclase activity, putative, expressed' |
| 43 | LOC_Os11g46070.1 | Inf | Inf | MLA10, putative, expressed |
| 44 | LOC_Os11g47000.1 | -4.29 | -4.58 | receptor-like protein kinase precursor, putative, expressed |
| 45 | LOC_Os11g47370.1 | -9.69 | -11.3 | 'protein\|expressed protein' |
| 46 | LOC_Os11g47400.1 | -7 | -5.82 |  |
| 47 | LOC_Os12g09190.1 | -1.46 | -1.62 | 'protein\|expressed protein' |
| 48 | LOC_Os12g10280.1 | Inf | Inf | aquaporin protein, putative, expressed |
| 49 | LOC_Os12g10320.1 | -1.53 | -1.88 |  |
| 50 | LOC_Os12g10330.1 | -3.49 | Inf | NB-ARC domain containing protein, expressed |
| 51 | LOC_Os12g10700.1 | -1.31 | -1.73 | expressed protein |
| 52 | LOC_Os12g10710.1 | -1.63 | -1.99 | NB-ARC domain containing protein, expressed |
| 53 | LOC_Os12g11370.1 | -5.94 | -4.57 | verticillium wilt disease resistance protein, putative, expressed |
| 54 | LOC_Os12g12010.1 | -6.45 | -4.28 | verticillium wilt disease resistance protein precursor, putative, expressed |
| 55 | LOC_Os12g12090.1 | Inf | Inf |  |
| 56 | LOC_Os12g12115.1 | Inf | Inf | 'protein\|expressed protein' |
| 57 | LOC_Os12g12120.1 | -3.09 | -3.75 | verticillium wilt disease resistance protein precursor, putative, expressed |
| 58 | LOC_Os12g12130.1 | Inf | Inf | verticillium wilt disease resistance protein, putative, expressed |
| 59 | LOC_Os12g12140.1 | Inf | Inf | 'protein\|expressed protein' |
| 60 | LOC_Os12g12560.1 | -2.41 | -2.49 | NADP-dependent oxidoreductase, putative, expressed |
| 61 | LOC_Os12g12570.1 | Inf | Inf | 'protein\|expressed protein' |
| 62 | LOC_Os12g12730.1 | -5.03 | -6.08 | OsCML28 - Calmodulin-related calcium sensor protein, expressed |
| 63 | LOC_Os12g13340.1 | -8.25 | -8.47 | expressed protein |
| 64 | LOC_Os12g13360.1 | Inf | Inf | cullin-1, putative, expressed |
| 65 | LOC_Os12g13674.1 | -6.48 | -5.29 | 'protein\|expressed protein' |
| 66 | LOC_Os12g13720.1 | -3.82 | -3.71 | Plant PDR ABC transporter associated domain containing protein, expressed |
| 67 | LOC_Os12g14059.1 | -3.12 | -3.44 | 'protein\|expressed protein' |
| 68 | LOC_Os12g14440.1 | -9.11 | -7.88 | Jacalin-like lectin domain containing protein, putative, expressed' |
| 69 | LOC_Os12g14840.1 | -8.11 | -9.07 | 'protein\|expressed protein' |
| 70 | LOC_Os12g15314.1 | Inf | Inf | staphylococcal nuclease homologue, putative, expressed |
| 71 | LOC_Os12g15505.1 | Inf | -6.14 | 'protein\|expressed protein' |
| 72 | LOC_Os12g18130.1 | Inf | Inf | 'protein\|expressed protein' |
| 73 | LOC_Os12g18360.1 | -3.53 | -4.13 | NB-ARC domain containing protein, expressed |
| 74 | LOC_Os12g18550.1 | Inf | Inf | 'protein\|expressed protein' |
| 75 | LOC_Os12g21784.1 | -10.43 | -9.4 | 'protein\|expressed protein' |
| 76 | LOC_Os12g23150.1 | -8.58 | -5.49 | 4-nitrophenylphosphatase-like, putative, expressed |
| 77 | LOC_Os12g23754.1 | Inf | Inf | 'protein\|expressed protein' |
| 78 | LOC_Os12g23780.1 | -8.23 | -9.19 | 'protein\|expressed protein' |
| 79 | LOC_Os12g24040.1 | -4.73 | -6.07 | glycosyl hydrolase family 9 protein, expressed |
| 80 | LOC_Os12g25160.1 | Inf | -5.16 | MATE, putative, expressed |
| 81 | LOC_Os12g32814.1 | -2.33 | -2.22 | pleiotropic drug resistance protein 3, putative, expressed |
| 82 | LOC_Os12g33130.1 | -3.21 | -3.01 | 'protein\|expressed protein' |
| 83 | LOC_Os12g37440.1 | -4.19 | -4.69 | 'protein\|conserved hypothetical protein' |
| 84 | LOC_Os12g40920.1 | -5.56 | -5.35 | bZIP transcription factor domain containing protein, expressed |
|  | Up regulated |  |  |  |
| 1 | LOC_Os01g04510.1 | 4.3 | 3.6 | 'protein\|expressed protein' |
| 2 | LOC_Os01g07170.1 | 2.6 | 2.6 | protein\|HORMA domain containing protein, putative, expressed' |
| 3 | LOC_Os01g49750.1 | 3.5 | 3.4 |  |
| 4 | LOC_Os02g06630.1 | 3.6 | 4.4 | peroxidase precursor, putative, expressed |
| 5 | LOC_Os02g50140.1 | Inf | Inf | caleosin related protein, putative, expressed' |
| 6 | LOC_Os02g50540.1 | 8.2 | 8.8 | 'protein\|expressed protein' |
| 7 | LOC_Os02g53200.1 | 1.7 | 1.6 | glucan endo-1,3-beta-glucosidase precursor, putative, expressed |
| 8 | LOC_Os02g57330.1 | 2.2 | 2.2 | expressed protein |
| 9 | LOC_Os02g57910.1 | Inf | Inf | OsFBX73 - F-box domain containing protein, expressed' |
| 10 | LOC_Os02g57924.1 | Inf | Inf | protein\|expressed protein' |
| 11 | LOC_Os02g58550.2 | 1.5 | 1.7 | chloroplast channel forming outer membrane protein, putative, expressed |
| 12 | LOC_Os03g12280.1 | 7 | 6.2 | 'protein\|expressed protein' |
| 13 | LOC_Os03g18779.1 | Inf | Inf |  |
| 14 | LOC_Os03g43100.1 | Inf | Inf | 'protein\|expressed protein' |
| 15 | LOC_Os04g02120.1 | 1.9 | 2.8 | 'protein\|expressed protein' |
| 16 | LOC_Os04g17660.2 | 3.5 | 3.1 | rhodanese-like domain containing protein, putative, expressed |
| 17 | LOC_Os05g12630.1 | 2.6 | 2.9 | 'protein\|expressed protein' |
| 18 | LOC_Os05g48450.5 | 1.3 | 1.5 | aminotransferase domain containing protein, putative, expressed |
| 19 | LOC_Os06g07020.1 | 2.2 | 2 | ZOS6-01 - C2H2 zinc finger protein, expressed |
| 20 | LOC_Os06g08041.1 | 1.4 | 1.5 | iron/ascorbate-dependent oxidoreductase, putative, expressed |
| 21 | LOC_Os06g17900.1 | 7.1 | 7.5 | disease resistance protein RPM1, putative, expressed' |
| 22 | LOC_Os07g03920.1 | 8.4 | 7 | lectin-like receptor kinase 7, putative, expressed' |
| 23 | LOC_Os07g06500.1 | 4.3 | 4.2 | protein\|OsFBL34 - F-box domain and LRR containing protein, expressed' |
| 24 | LOC_Os07g08934.1 | Inf | Inf | 'protein\|expressed protein' |
| 25 | LOC_Os07g11490.1 | 3.1 | 3.6 | 'protein\|expressed protein' |
| 26 | LOC_Os07g25550.1 | Inf | Inf | hAT dimerisation domain-containing protein, putative, expressed' |
| 27 | LOC_Os07g25590.1 | 1.3 | 1.4 | decarboxylase, putative, expressed |
| 28 | LOC_Os07g26100.1 | 7.8 | 7.8 | 'protein\|expressed protein' |
| 29 | LOC_Os07g27350.1 | 1.2 | 1.1 | atuA, putative, expressed |
| 30 | LOC_Os07g30240.1 | 3.4 | 3.7 | mutS family domain IV containing protein, expressed |
| 31 | LOC_Os07g38240.1 | 2.8 | 2.6 | ZOS7-05 - C2H2 zinc finger protein, expressed |
| 32 | LOC_Os07g38250.1 | 7 | 7 | expressed protein |
| 33 | LOC_Os08g09610.1 | 9.7 | 10 | 'protein\|expressed protein' |
| 34 | LOC_Os08g10250.1 | 4.2 | 3.8 | SHR5-receptor-like kinase, putative, expressed |
| 35 | LOC_Os08g10340.1 | 2.3 | 2.4 | 'protein\|OsFBX278 - F-box domain containing protein, expressed' |
| 36 | LOC_Os08g12850.1 | 1.6 | 1.8 | pentatricopeptide, putative, expressed |
| 37 | LOC_Os08g13070.1 | 7.5 | 8.1 | MBTB23 - Bric-a-Brac, Tramtrack, Broad Complex BTB domain with Meprin and TRAF Homology MATH domain, expressed' |
| 38 | LOC_Os08g13280.1 | 5.9 | 5.9 | 'protein\|expressed protein' |
| 39 | LOC_Os08g13430.1 | 10.1 | 10 |  |
| 40 | LOC_Os08g13699.1 | 7.1 | 7.3 | 'protein\|expressed protein' |
| 41 | LOC_Os08g13795.1 | 6.3 | 6.7 | 'protein\|expressed protein' |
| 42 | LOC_Os08g13905.1 | 7.7 | 7.8 | 'protein\|expressed protein' |
| 43 | LOC_Os08g13990.1 | 2.4 | 2.4 | pentatricopeptide, putative, expressed |
| 44 | LOC_Os08g14940.1 | 2.6 | 2.7 | receptor kinase, putative, expressed |
| 45 | LOC_Os08g16350.1 | 6.7 | 6.8 | 'protein\|expressed protein' |
| 46 | LOC_Os08g17370.1 | Inf | Inf | transmembrane 9 superfamily member, putative, expressed' |
| 47 | LOC_Os08g17655.1 | 6.3 | 6.1 | 'protein\|expressed protein' |
| 48 | LOC_Os08g18079.1 | 6.1 | 6.3 | 'protein\|expressed protein' |
| 49 | LOC_Os08g19374.4 | Inf | Inf | 'protein\|expressed protein' |
| 50 | LOC_Os08g20090.2 | Inf | Inf | 'protein\|expressed protein' |
| 51 | LOC_Os08g20660.1 | 0.9 | 0.8 | sucrose-phosphate synthase, putative, expressed |
| 52 | LOC_Os08g20680.1 | 8.1 | 8.3 | 'protein\|expressed protein' |
| 53 | LOC_Os08g21879.1 | 4.4 | 4.8 | 'protein\|expressed protein' |
| 54 | LOC_Os08g23280.1 | Inf | Inf | 'protein\|expressed protein' |
| 55 | LOC_Os08g26700.1 | 3.7 | 3.8 | 'protein\|expressed protein' |
| 56 | LOC_Os08g26710.1 | 8.6 | 8.8 |  |
| 57 | LOC_Os08g27540.3 | Inf | Inf | 'protein\|expressed protein' |
| 58 | LOC_Os08g27580.4 | 4.9 | 5 | protein\|expressed protein |
| 59 | LOC_Os08g27720.1 | 2.3 | 2.6 | pirin, putative, expressed |
| 60 | LOC_Os08g28670.1 | 3.2 | 4.2 | 'protein\|pathogenesis-related Bet v I family protein, putative, expressed' |
| 61 | LOC_Os08g28790.1 | Inf | Inf | dirigent, putative, expressed |
| 62 | LOC_Os08g29020.1 | 5.3 | 4.7 | wall-associated kinase-like 2, putative, expressed |
| 63 | LOC_Os08g30370.1 | Inf | Inf | 'protein\|expressed protein' |
| 64 | LOC_Os08g30554.1 | Inf | Inf | 'protein\|expressed protein' |
| 65 | LOC_Os08g30590.1 | 3.6 | 3.7 | C1-like domain containing protein, expressed |
| 66 | LOC_Os08g31440.1 | Inf | Inf | 'protein\|expressed protein' |
| 67 | LOC_Os08g31569.1 | Inf | Inf | 'protein\|expressed protein' |
| 68 | LOC_Os08g31670.1 | 5.5 | 6.2 | transporter, putative, expressed |
| 69 | LOC_Os08g34830.1 | Inf | Inf | hAT dimerisation domain-containing protein, putative, expressed' |
| 70 | LOC_Os08g38910.2 | 1.5 | 1.8 | CAMT_MESCR Caffeoyl-CoA |
| 71 | LOC_Os09g14530.1 | 6.4 | 5.9 | hAT dimerisation domain-containing protein, putative |
| 72 | LOC_Os09g29270.1 | 9.1 | 8.6 | 'protein\|expressed protein' |
| 73 | LOC_Os10g01380.1 | 6.8 | 6.9 |  |
| 74 | LOC_Os10g04625.1 | 4.4 | 4.2 | 'protein\|expressed protein' |
| 75 | LOC_Os11g10850.1 | 4.4 | 4.6 | dirigent, putative, expressed |
| 76 | LOC_Os11g33240.1 | 1 | 1.2 | citrate synthase, putative, expressed |
| 77 | LOC_Os11g34130.1 | 2.3 | 2.5 | expressed protein |
| 78 | LOC_Os11g34210.2 | 1.4 | 1.7 | aspartyl/glutamyl-tRNA amidotransferase subunit B, putative, expressed' |
| 79 | LOC_Os11g34220.1 | Inf | Inf | 'protein\|expressed protein' |
| 80 | LOC_Os11g34824.1 | 8.6 | 8.8 | 'protein\|expressed protein' |
| 81 | LOC_Os11g42030.1 | Inf | Inf | expressed protein' |
| 82 | LOC_Os11g42040.1 | 9.3 | 9.5 | non-TIR-NBS-LRR type resistance protein, putative, expressed |
| 83 | LOC_Os11g42070.1 | Inf | Inf | Leucine Rich Repeat family protein, expressed' |
| 84 | LOC_Os11g43404.1 | 2.4 | 2.7 | 'protein\|expressed protein' |
| 85 | LOC_Os11g44800.1 | 6.5 | 6.5 | 'protein\|expressed protein' |
| 86 | LOC_Os11g44870.1 | 1.4 | 2.2 | expressed protein |
| 87 | LOC_Os11g45050.1 | 1.5 | 1.5 | NBS-LRR disease resistance protein, putative, expressed |
| 88 | LOC_Os11g45930.1 | 2.4 | 2.2 | NBS-LRR type disease resistance protein, putative, expressed |
| 89 | LOC_Os11g46210.1 | Inf | Inf | NB-ARC domain containing protein, expressed' |
| 90 | LOC_Os11g46870.1 | 4.8 | 4.5 | protein kinase, putative, expressed |
| 91 | LOC_Os11g46900.1 | 9.4 | 8.3 | wall-associated receptor kinase 3 precursor, putative, expressed |
| 92 | LOC_Os11g47140.1 | 8.2 | 6.8 | OsWAK123 - OsWAK receptor-like protein kinase, expressed' |
| 93 | LOC_Os11g47330.1 | 5 | 5.3 | ATP-grasp domain containing protein, expressed |
| 94 | LOC_Os11g47910.1 | 1.6 | 1.4 | SCARECROW, putative, expressed |
| 95 | LOC_Os11g48110.1 | Inf | Inf | phenylalanine ammonia-lyase, putative, expressed' |
| 96 | LOC_Os12g08240.1 | 4.2 | 3.7 |  |
| 97 | LOC_Os12g08250.1 | Inf | Inf | 'protein\|expressed protein' |
| 98 | LOC_Os12g10870.1 | 5.7 | 5.8 | verticillium wilt disease resistance protein, putative, expressed |
| 99 | LOC_Os12g11830.1 | 2.8 | 3.2 | 'protein\|expressed protein' |
| 100 | LOC_Os12g11860.1 | 9.3 | 9.1 | verticillium wilt disease resistance protein precursor, putative, expressed' |
| 101 | LOC_Os12g12765.1 | 7.9 | 8 | 'protein\|expressed protein' |
| 102 | LOC_Os12g16240.1 | 2.6 | 2.3 | nmrA-like family domain containing protein, expressed |
| 103 | LOC_Os12g17410.1 | 4.9 | 4.9 | NB-ARC domain containing protein, expressed |
| 104 | LOC_Os12g17480.1 | 2.5 | 2.7 | MLA12, putative, expressed |
| 105 | LOC_Os12g20410.1 | 1.2 | 1.2 | protein\|matrix attachment region binding protein, putative, expressed' |
| 106 | LOC_Os12g21950.1 | Inf | Inf | 'protein\|expressed protein' |
| 107 | LOC_Os12g33160.1 | 3.8 | 3.5 | RGH1A, putative, expressed' |
| 108 | LOC_Os12g33180.1 | 4.1 | 4.2 | 'protein\|pnn protein, putative, expressed' |
| 109 | LOC_Os12g33194.1 | Inf | Inf | \|expressed protein' |
| 110 | LOC_Os12g33300.1 | 3.7 | 3.1 | integral membrane protein DUF6 containing protein, expressed |
| 111 | LOC_Os12g33610.1 | 8.7 | 8.8 | phenylalanine ammonia-lyase, putative, expressed |
| 112 | LOC_Os12g34510.1 | 3.5 | 3.7 | Core histone H2A/H2B/H3/H4 domain containing protein, putative, expressed |
| 113 | LOC_Os12g34796.1 | 3.4 | 3.6 |  |
| 114 | LOC_Os12g35590.1 | 10 | 10.2 | 'protein\|expressed protein' |
